# Analysis of 142 genes resolves the rapid diversification of the rice genus

**DOI:** 10.1186/gb-2008-9-3-r49

**Published:** 2008-03-03

**Authors:** Xin-Hui Zou, Fu-Min Zhang, Jian-Guo Zhang, Li-Li Zang, Liang Tang, Jun Wang, Tao Sang, Song Ge

**Affiliations:** 1 State Key Laboratory of Systematic and Evolutionary Botany, Institute of Botany, Chinese Academy of Sciences, Beijing, 100093, China; 2 Beijing Genomics Institute, Beijing, 101300, China; 3 Department of Plant Biology, Michigan State University, East Lansing, MI 48824, USA; 4 The Graduate School, Chinese Academy of Sciences, Beijing, 100039, China

## Abstract

**Background:**

The completion of rice genome sequencing has made rice and its wild relatives an attractive system for biological studies. Despite great efforts, phylogenetic relationships among genome types and species in the rice genus have not been fully resolved. To take full advantage of rice genome resources for biological research and rice breeding, we will benefit from the availability of a robust phylogeny of the rice genus.

**Results:**

Through screening rice genome sequences, we sampled and sequenced 142 single-copy genes to clarify the relationships among all diploid genome types of the rice genus. The analysis identified two short internal branches around which most previous phylogenetic inconsistency emerged. These represent two episodes of rapid speciation that occurred approximately 5 and 10 million years ago (Mya) and gave rise to almost the entire diversity of the genus. The known chromosomal distribution of the sampled genes allowed the documentation of whole-genome sorting of ancestral alleles during the rapid speciation, which was responsible primarily for extensive incongruence between gene phylogenies and persisting phylogenetic ambiguity in the genus. Random sample analysis showed that 120 genes with an average length of 874 bp were needed to resolve both short branches with 95% confidence.

**Conclusion:**

Our phylogenomic analysis successfully resolved the phylogeny of rice genome types, which lays a solid foundation for comparative and functional genomic studies of rice and its relatives. This study also highlights that organismal genomes might be mosaics of conflicting genealogies because of rapid speciation and demonstrates the power of phylogenomics in the reconstruction of rapid diversification.

## Abstract 2

The relationships among all diploid genome types of the rice genus were clarified using 142 single-copy genes

## Background

Rice is one of the most important crops in the world, providing the staple food for more than one-half of the world's population [1,2]. The completion of rice genome sequencing has made rice and its wild relatives an increasingly attractive system for biological studies at the genomic level [3-5]. Considerable insights have been recently gained into comparative genomics between rice and other cereal crops of the grass family [6] and between the species of the rice genus, *Oryza *[7,8]. To take full advantage of rice genome resources for basic biological research and rice breeding, we will benefit from the availability of a robust phylogeny of the rice genus.

The genus *Oryza *consists of 2 cultivated and approximately 22 wild species distributed in a diverse range of habitats in tropics and subtropics of the world [9]. By assessing the degree of meiotic pairing in interspecific hybrids, traditional genome analyses grouped the majority of *Oryza *species into five diploid and two allotetraploid genome types: A-, B-, C-, E-, F-, BC-, and CD-genomes [10]. Because of the difficulties in obtaining hybrids with presumably more distantly related species, three additional genomes, G-, HJ-, and HK-genomes, were later recognized based on total genomic DNA hybridization [11] and molecular phylogenetics [12]. In *Oryza*, one-third of extant species are allotetraploids that originated through hybridization between diploid genomes, and, in particular, four (B-, E-, F-, and G-genomes) out of the six diploid genomes each have a single species [1,9,13]. Consequently, elucidating the phylogenetic relationships of the diploid rice genomes is critically important for understanding the evolutionary history of the entire genus.

Despite extensive studies on evolutionary relationships among rice genomes and species [10,12,14-16], the phylogenetic relationships among genomes remained elusive until a study that sampled all recognized *Oryza *species and utilized sequences of two nuclear and one chloroplast genes [12]. This study supported the monophyly of each of the previously recognized genome types and reconstructed the origins of tetraploid species. Nevertheless, two areas of the phylogeny were left unresolved due to incongruence between gene trees. These included the relationship among A-, B-, and C-genomes and that among the F-genome, G-genome, and the rest of the genus [12]. The incongruence was highlighted in the rice phylogenetic literature, where all three possible relationships among A-, B-, and C-genomes were suggested [10,12,16-18]. More remarkable is the position of the F-genome, which varied from being the most basal lineage of the entire genus [16,19] to being nested within the recently diverged A-genome [15,20].

The recent decade has witnessed the successful utilization of large quantities of DNA sequences in solving long-standing phylogenetic problems [21-30]. As a growing number of genomes are decoded, phylogenetic reconstruction using genome-wide markers, or phylogenomics [31,32], will provide unprecedented opportunities to elucidate the previously controversial evolutionary relationships at all taxonomic levels [31,33]. In this study, we screened the genome sequences of two rice cultivars and sampled 142 single-copy genes as markers for reconstructing the phylogeny of all diploid rice genomes. This phylogenomic analysis, for the first time, fully resolved the relationships of the rice genome types. It further revealed that two episodes of rapid diversification in the rice genus were responsible for the phylogenetic incongruence that persisted in the previous studies. We suggest that rapid diversification might be widespread in organismal evolution and caution that under rapid speciation, large data sets or phylogenomic approach are required to resolve phylogenetic relationships with a high degree of confidence.

## Results

### Phylogeny inferred from concatenated sequences of 142 genes

After an extensive screen of rice genome sequences, we identified and sequenced 142 single-copy genes that were most likely free of the paralogy problem for reconstructing the phylogeny of all diploid genome types of *Oryza *(Table 1; see Materials and methods for details of gene screening). These genes are distributed throughout the 12 rice chromosomes and represent a genome-wide sampling of phylogenetic markers (Additional data files 1 and 2). After removing regions with ambiguous alignment, we concatenated the 142 genes into a data matrix of 124,079 bp, with exons accounting for 43% of the total sequence. The concatenated alignment contained 26,838 (21.6%) variable sites, of which 6,753 (5.4%) were phylogenetically informative (Additional data file 2).

**Table 1:** Information on the materials used in this study

Species	Genome	Accession number*	Origin	No. of genes sequenced	No. of sites aligned
*Oryza sativa*^†^	A	93-11	China	62	52,092
*O. rufipogon*	A	105480	India	142	124,079
*O. barthii*^†^	A	104132	Cameroon	62	52,092
*O. punctata*	B	103903	Tanzania	141	124,079
*O. officinalis*	C	104972	China	142	124,079
*O. rhizomatis*^†^	C	103410	Sri Lanka	62	52,092
*O. eichingeri*^†^	C	105415	Sri Lanka	62	52,092
*O. australiensis*	E	105263, 101410	Australia	135	124,079
*O. brachyantha*	F	105151	Sierra Leone	124	124,079
*O. granulata*	G	M8-15, 106469	China, Vietnam	124	124,079
*Leersia tisserantti*	-	105610	Cameroon	122	124,079

*All accession numbers were obtained from the International Rice Research Institute at Los Banos, Philippines, except for M8-15, which was collected by the authors. ^†^Sixty-two genes were sequenced for these species and used only for testing the effect of dense sampling. Sequences of *O. sativa *(*93-11*) were retrieved from the BGI-RIS database.

**Figure 1:**
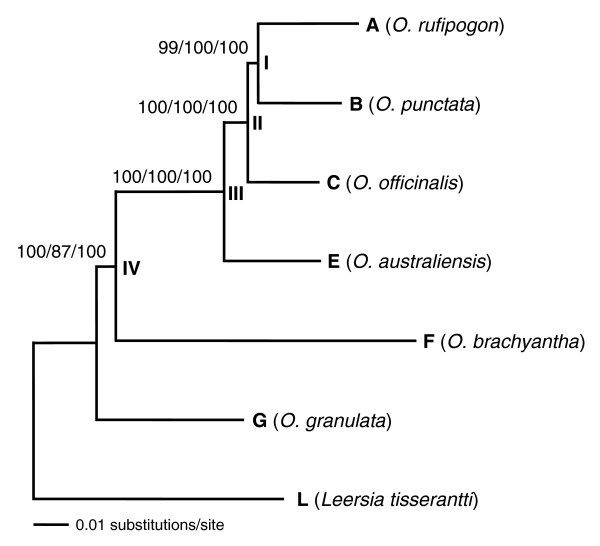
ML tree inferred from the concatenated sequences of 142 genes using the GTR+Γ model. The same topology was obtained from MP and BI. The letters A, B, C, E, F, and G represent all recognized diploid genome types of *Oryza*, and L represents the outgroup. The names of the species that represent the genome types and outgroup are in parentheses. Numbers above branches indicate bootstrap support of ML and MP, and posterior probability of BI, respectively. Four internal branches of *Oryza *genome types are indicated with I, II, III, and IV. Branch length is proportional to the number of substitutions measured by the scale bar.

Phylogenetic analyses of the concatenated sequences using maximum likelihood (ML), maximum parsimony (MP) and Bayesian inference (BI) all yielded a single fully resolved tree with high bootstrap support or Bayesian posterior probability (PP) for all internal branches (Figure 1). We labeled these branches as I, II, III, and IV. The relationships between A-, B-, and C-genomes are finally resolved, with the sister relationship between A- and B-genomes supported by 99-100% bootstrap support or PP. The F-genome, which jumped all over the previously reported phylogenies, is firmly placed between the basal G-genome and the rest of the genome types.

Because the increase in sequence length or the number of sampled genes does not guarantee the elimination of systematic errors [28,34,35], it is necessary to investigate the potential impact of systematic bias on our phylogenetic reconstruction. First, we tested homogeneity of base composition across species for total, intron, exon, and three codon sites of the concatenated data set. The results indicated that four nucleotide bases occurred in almost equal proportions and the GC content varied little among species for all data partitions (χ^2 ^tests, *P *= 0.346-1.0; Additional data file 3). The potential compositional bias was also examined with analysis using log-determinant (LogDet) distance [36]. This yielded the same topology as ML, MP, and BI (Table 2). These tests suggest that the concatenated data set did not contain compositional signals that could have biased the phylogenetic reconstruction.

**Table 2:** Bootstrap support from 1,000 replicates for the four internal branches of phylogenetic trees based on the concatenated sequences using different methods

	Bootstrap support (%)			
	
Method	I	II	III	IV
RY-coding strategy (ML)	93	100	100	73
LogDet distance (NJ)	93	100	100	90
Gene bootstrap (ML)	72	100	100	88

NJ, neighbor-joining.

Second, we analyzed rate constancy among lineages using Tajima's relative rate test [37]. When the concatenated sequences were considered, results showed that the null hypothesis of rate constancy was rejected in almost all pairs of contrasts (*P *< 0.01). It is noteworthy that the F-genome evolved at a faster rate and the G-genome evolved at a slower rate than other genomes (Additional data file 4). To explore the potential impact of rate heterogeneity on tree reconstruction, we adopted the RY-coding strategy that discards fast-evolving transitions and consequently makes phylogenetic reconstructions less susceptible to uneven occurrence of multiple hits among lineages [34,38]. The tree obtained from the re-coded data set was topologically identical to that shown in Figure 1 (Table 2). To further test the potential long-branch attraction effect of the fast-evolving F-genome, we identified genes that evolved more rapidly in the F-genome than in the A-, B-, and C-genomes. We calculated the ratio of the mean distance between the F-genome and each of A-, B-, and C-genomes to the mean distances among A-, B-, and C-genomes for each gene. We then progressively excluded fast-evolving genes of the F-genome in a decreasing order of the ratios. The topology based on the remaining genes did not change until more than 50 genes were excluded (Additional data file 5). These suggest that rate heterogeneity was not severe enough to cause significant systematic bias.

Third, to examine the potential systematic errors caused by model misspecification [30,32,35], we applied a series of homogeneous and mixed models in BI and evaluated the relative merits of competing models by Bayes factors. Although Bayes factor comparisons showed that all mixed models outperformed the homogeneous models significantly (Additional data files 6 and 7), analyses with all 14 alternative models, including ones incorporating the covarion model, which accounts for heterotachous signal, yielded the same topology as shown in Figure 1, with all internal branches supported by 100% PP. Taken together, the above analyses indicate that the phylogeny inferred from the concatenated gene sequences was not biased by systematic errors.

We next tested whether the resulting phylogeny could have been influenced by a subset of the genes. A gene bootstrap analysis was performed with 1,000 replicates [27]. For each replicate, we randomly drew 142 genes with replacement from the entire pool. The sampled genes were concatenated and analyzed using ML. The results strongly supported the topology shown in Figure 1 (Table 2), indicating that the phylogenetic reconstruction was not dominated by a subset of the 142 genes.

**Figure 2:**
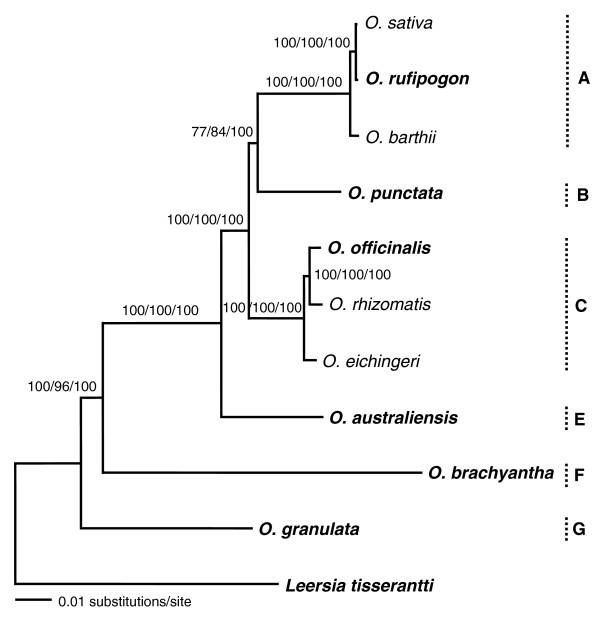
ML tree inferred based on concatenation of 62 genes from 11 species using the GTR+Γ model. Numbers above branches indicate bootstrap support of ML and MP, and posterior probability of BI analyses, respectively. Capital letters (A to G) beside the tree specify the genome type of the species. For the species in bold, 142 genes were sequenced and used in the analyses as shown in Figure 1.

Finally, we investigated whether within-genome sampling would influence the phylogenetic reconstruction. Because the A- and C-genomes have more than one species, while each of the remaining genomes has only one species [1,9,13], we sequenced 62 genes for an additional A-genome species, *O. barthii*, and two additional C-genome species, *O. rhizomatis *and *O. eichingeri *(Table 1). Sequences of cultivated rice, *O. sativa*, belonging to the A-genome were also retrieved from the BGI-RIS Database [39] and added to the data set. Phylogenetic analyses of the 11 species generated the same inter-genome relationship as the one shown in Figure 1 (Figure 2). This indicates that one species sampled from each genome was sufficiently representative for the reconstruction of the genome relationships.

### Phylogenetic incongruence and network analyses

**Figure 3:**
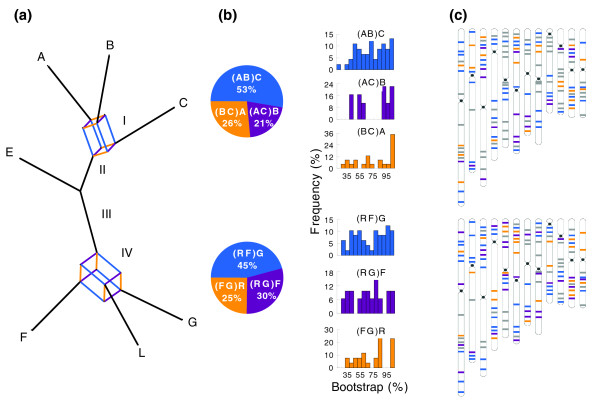
Genome-wide incongruence. A, B, C, E, F, and G represent *Oryza *genome types and L represents the outgroup, *Leersia*. **(a) **Consensus network constructed from ML trees at a threshold of 0.15. The two boxes indicate the relatively high levels of incongruence among gene trees associated with internal branches I and IV. Branch length is proportional to the frequency of occurrence of a particular split of all gene trees. R represents the rest of the genome types, including A-, B-, C-, and E-genomes. Color schemes: for the box associated with branch I, blue, orange, and purple illustrate splits supporting alternative topologies, (AB)C, (BC)A, and (AC)B, respectively; for the box associated with branch IV, blue, orange, and purple illustrate splits supporting alternative topologies, (RF)G, (FG)R, and (RG)F, respectively. **(b) **Pie graphs indicate the proportions of gene trees that support alternative splits in the corresponding boxes at the left. Histograms at the right illustrate the distribution of ML bootstrap support for the corresponding split (in the corresponding colors). **(c) **Illustration of the relative physical locations of the 142 sampled genes on the 12 rice chromosomes based on rice genome sequences. The colors indicate genes supporting a split or topology coded in the same color in the corresponding boxes on the consensus network. Genes coded in gray are those that had no input in the topology illustrated in the pie graphs and those not included for the construction of the consensus network because of missing data.

When phylogenetic analyses were done for each of the 142 genes separately, more than 40 different optimal trees were generated, indicative of extensive incongruence among gene phylogenies. To gain insight into the extent of incongruence, we constructed consensus networks from ML trees of the 106-gene data set without missing data. Figure 3a shows the network at a threshold of 0.15, which presents branches appearing at a frequency of 15% or higher of all gene trees. Two boxes are evident in the network, indicating that topological incongruence is concentrated on branch I involving the relationships between A-, B-, and C-genomes and branch IV involving the relationships between the F-genome, G-genome, and the rest of the genome types (R). We also explored the features of consensus networks by increasing the threshold from 0.05 and found that the boxes were collapsed when the threshold reached 0.3 and it ended up with the topology identical to that shown in Figure 1 (Additional data file 8). These results further support the phylogenetic relationships revealed by the concatenated data set and highlight the incongruence involving branches I and IV.

In the first box, the length of parallel edges supporting split AB|CEFGL is longer than those supporting splits AC|BEFGL and BC|AEFGL (Figure 3a), suggesting that a higher proportion of consensus signal groups A and B together. This is in agreement with the result that a larger number of gene trees (53%) support the sister relationship of A and B than those supporting the alternative sister relationship between B and C (26%) or between A and C (21%) (Figure 3b). For the second box, the length of parallel edges supporting split ABCEF|GL is longer than those supporting the two alternative splits. This is also consistent with the result that 45% of gene trees support the sister relationship between R and F while 30% and 25% of gene trees support the sister relationships between R and G or between F and G, respectively (Figure 3b).

To further explore the incongruence among gene trees, we performed the incongruence length difference (ILD) test based on two partitioning strategies and found that there was no significant incongruence between any pair of the process partitions (intron and three codon positions; Additional data file 9). In contrast, significant heterogeneity was found among gene partitions, including tests among all gene partitions as a whole (*P *< 0.01) and between pairwise comparisons and between each gene and the remaining genes combined (Additional data file 10). These results were consistent with the distributions of bootstrap support for alternative topologies at the two boxes (Figure 3b). For each box, there is a substantial proportion of high bootstrap support for alternative topologies, suggesting that the competing topologies are well supported on the respective gene trees. Remarkably, genes supporting any given topology are distributed randomly among the 12 chromosomes (χ^2 ^test, *P *= 0.233-0.823), indicative of a genome-wide incongruence (Figure 3c).

To address the question of whether the incongruence among genes is attributed to different evolutionary histories of genes or merely systematic errors [40,41], we conducted tests for systematic bias for each of the 142 genes. The Chi-square test revealed that there was no heterogeneity of base composition for any gene. However, rate heterogeneity was detected for some genes by the relative rate test. We then conducted phylogenetic analyses for each gene using different strategies, including ML, MP, RY-coding, and LogDet distance. The comparison of bootstrap 75% majority-rule consensus trees showed that only 4 out of 142 genes yielded incompatible topologies between different methods of analyses (Additional data file 11). This indicates that there are few systematic errors involved in individual genes and the incongruence among gene partitions is governed mainly by different evolutionary histories of genes.

### Short branches and their resolution

Different evolutionary histories of genes can be attributed to three major factors, including paralogy, hybridization, and lineage sorting [40]. We have largely ruled out the potential effect of paralogy by carefully screening gene markers (see Materials and methods). The pattern of incongruence also does not support hybrid speciation because hybridization would have led to two major incongruent topologies rather than the presence of a leading topology with two alternative topologies occurring at nearly equal frequencies for both clades I and IV. The random distribution on chromosomes of the genes that support a given topology (Figure 3c) does not support the hybridization hypothesis either because related or linked loci should share gene trees if the species have a history of introgression or hybridization [42]. Therefore, we are left with the hypothesis of lineage sorting as the primary explanation for the incongruence.

Population genetic theory suggests that lineage sorting is more likely to occur at an internal branch of a species tree that is short (few in generations) and wide (large in effective population sizes) [43,44]. Based on estimation by the ML method, branches I and IV were the shortest internal braches on the concatenation tree and obtained relatively low support values in analyses with different methodologies (Figure 1 and Table 2). For branch I, there is a sufficient amount of published data that allow us to estimate the probability to obtain the species tree from a given gene. That is, *P *= 1 - 2/3exp(-*t*) under the coalescent model, where *t *is the time between two speciation events in the unit of generations/2*Ne *and *Ne *is the effective population size [43].

Using the previously reported nucleotide diversity at silent sites (θ_sil _= 0.0038-0.0095) for the A- and C-genome species [45,46] and a substitution rate for grasses (5.9 × 10^-9 ^substitutions per synonymous site per year) [47,48], we estimated that the effective population sizes of these *Oryza *species ranged from 1.6 × 10^5 ^to 4.0 × 10^5^. A speciation model test on three C-genome species suggested that their ancestral population sizes were approximately ten-fold larger than those of each species [45]. Thus, the ancestral population size of A-, B-, and C-genomes (*Ne*) should be at least 1.6 × 10^6^. Because the A-genome species began to diverge approximately 2 Mya [49] and divergence between B- and C-genomes occurred approximately 3.8 Mya [45], the time between two speciation events should be less than 1.8 million years. Given the generation time of 1-2 years in wild rice species [50], the number of generations between the two speciation events is at most 1.8 × 10^6^. The estimated upper limit of generations together with the lower limit of *Ne *led to the calculation of the upper limit of *P *as 0.62. This implies that there is less than a 62% chance for any given gene tree to be the same as the species tree or less than 62% of gene trees from the sampled genes will be congruent with the species tree. Our finding that 53% of gene trees support the sister relationship of A- and B-genomes agrees with the theoretical expectation (Figure 3b), which further supports the lineage sorting hypothesis.

For branch IV, the divergence happened at greater depth in the tree and thus homoplasy resulting from mutational saturation might be a factor to cause incongruent gene phylogenies [33,51]. However, analyses of saturation plots did not reveal any mutational saturation for the concatenated data set (Additional data file 12), suggesting that lineage sorting is still the most plausible explanation for the incongruence.

**Figure 4:**
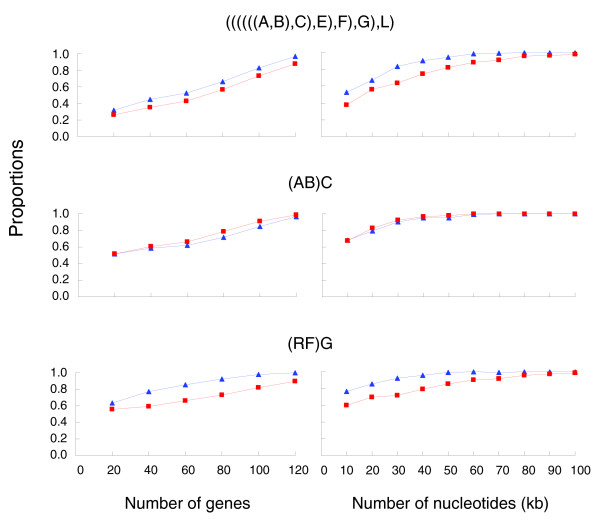
The proportions of topologies (or clades) that are identical to those shown in Figure 1 based on resampling of 142 gene sequences at various scales. Results of ML and MP analyses are indicated by blue and red, respectively. Genome types are represented with the same capital letters as in Figure 3.

To assess how much of the data set might be needed to resolve such short branches, we explored the relationship between the number of genes or nucleotide sites and the proportion of gene trees that support the topology or clades shown in Figure 1. The results demonstrated that the probability of getting identical topology or clades as in Figure 1 steadily increased with the number of genes or sites sampled, regardless of methods used, although ML generally performed better than MP (Figure 4). Using 95% of identical gene trees or clades in 500 replicates as a criterion, about 120 genes were needed for both ML and MP methods to resolve branch I and more than 80 (ML) and 120 (MP) genes were needed to resolve branch IV. Additionally, 120 (ML) or more genes (MP) were needed to resolve both branches simultaneously (Figure 4 and Additional data file 13).

When nucleotide sites rather than genes were the unit of resampling, about 40 kb of nucleotides were sufficient to resolve branch I with both methods. This is equivalent to 46 sampled genes in length given the average length of 874 bp per gene. It took approximately 40 kb and 80 kb (approximately 92 genes) for ML and MP, respectively, to resolve branch IV. A total of 50 kb (approximately 57 genes) for ML and 80 kb for MP were sufficient to resolve both branches simultaneously (Figure 4 and Additional data file 13). These results indicate that random sampling of unlinked nucleotides has a higher power of phylogenetic resolution than sampling contiguous nucleotides such as those within a gene.

## Discussion

This study fully resolved the phylogeny of the rice genomes. Through extensive tests and analyses, we demonstrate that the phylogenetic reconstruction based on the sequences of 142 genes was not biased by systematic or sampling errors and was insensitive to phylogenetic methods or model specification. We identified across the genome a remarkable level of incongruence of gene phylogenies at the two shortest internal branches (Figures 1 and 3). Our analyses clearly indicated that lineage sorting was a primary cause for the difficulty of resolving two branches of the rice phylogeny that underwent rapid diversification. Even more remarkably, lineage sorting occurred for genes distributed randomly across all 12 rice chromosomes (Figure 3c). This study thus documents a case of genome-wide lineage sorting that gave rise to species with the mosaic of ancestral genomes [26,52]. One implication of our findings is that special caution must be taken in interpreting phylogenetic relationships of rapidly diverged lineages even though the relationships are strongly supported on a single gene phylogeny. Our results also imply that although it may not be feasible to have a large number of genes to resolve a short branch for groups with limited genomic resources, utilization of a few genes should provide a clue to the extent to which lineage sorting may lead to erroneous phylogenies [12].

The biological implications for the presence of two short branches (I and IV) that reflect two episodes of rapid diversification of the rice genus are profound. Based on a molecular clock estimate, the first event occurred approximately 10 Mya [53] and led to a rapid diversification of the G-genome, F-genome and a lineage that subsequently diversified into the rest of the rice genomes. Additionally, the H-, J-, and K-genomes that are now only present in extant tetraploid species, including *O. longiglumis *and *O. ridleyi *with the HJ-genome and *O. schlechteri *and *O. coarctata *with the HK-genome, also diverged around this time [12,53]. The second event led to the diversification of A-, B-, and C-genomes approximately 5 Mya [45,53]. Therefore, the two episodes of rapid diversification gave rise to almost the entire diversity of the genus. Because the *Oryza *species are distributed in distinct habitats across four continents [1,50], it would be interesting to further investigate whether the rapid diversification was coupled with adaptive radiation under certain geological and ecological conditions [54,55].

Rapid speciation, particularly ancient radiation, featured by the short internal branches in phylogenetic trees, poses an extraordinary challenge to systematic and evolutionary biologists [33,51,55,56]. It has been observed at a variety of time depths ranging from as early as the Cambrian explosion of animal phyla over 550 Mya [25] to as recent as the divergence between human, chimpanzee, and gorilla a few Mya [52,57,58]. In many cases, phylogenetic relationships seemed to be an irresolvable polytomy [23,56,59] because of the rapid radiations. Such closely spaced series of speciation events was accordingly considered to be "bushes in the Tree of Life" [33]. To date, rapid evolutionary radiations have been proposed to be the most plausible explanation for the poorly resolved phylogenies or polytomies in many organisms such as aphids, black flies, bees, birds, turtles, mammals, and higher plants [29,30,33,51,60]. However, a growing body of evidence showed that many assumed polytomies were 'soft' and could be resolved into sequential bifurcations with additional data and proper methods of phylogenetic analysis [52,59,61,62]. In a study of phylogenetic relationships among tetrapod, coelacanth, and lungfish, Takezaki *et al*. [59] obtained an irresolvable trichotomy although sequences of 44 nuclear genes were analyzed. Using computer simulation, they concluded that more than 200 loci would have to be analyzed to resolve the relationships among the three lineages if the fish-to-tetrapod transition interval was 10-20 million years long. The once unresolved relationship among human, chimpanzee, and gorilla is a typical example of soft polytomies. Recent analyses with an increased amount of molecular data resolved human and chimpanzee into a sister group [52,57,58]. Our results exemplify that rapid speciation within an angiosperm genus can be reliably resolved as long as a sufficient amount of unlinked DNA sequences is available.

However, we should also realize that the increase in the amount of data alone may not provide a universal solution to all short branches on the Tree of Life. It is theoretically possible that certain branches are not resolvable even with whole genome sequences if time intervals between speciation were extremely short and the speciation events were sufficiently ancient [31,33,51]. These branches are considered to be 'hard' polytomies [33,61]. Nevertheless, both soft and hard polytomies provide historical information on evolutionary processes and a phylogenetic analysis with genome-wide information can be most helpful for understanding the evolutionary histories behind these seemingly problematic, but perhaps intriguing, branches of the Tree of Life.

For soft polytomies, an obviously interesting question is how many DNA sequences would be needed to resolve rapid speciation considering that DNA sequences have been, and will remain, major sources of biological data [31,32]. The mosaic genome or different evolutionary histories of genes under rapid speciation, in conjunction with other factors associated with species divergence (for example, selection and high homoplasy of ancient speciation [33,51]), brings about difficulties in resolving speciation events when using a small number of regions/genes or limited characters [22,59]. This study shows that as many as 120 genes with an average length of 874 bp or 50 kb of randomly sampled nucleotides from 142 genes are needed to resolve clades I and IV simultaneously with over 95% confidence (Figure 4). Clearly, blocks of contiguous nucleotide sites were less powerful in phylogenetic resolution than samples consisting of sites drawn randomly from the genome because nucleotides within genes do not evolve independently [22,63]. This implies that for the same amount of sequence data, a larger number of unlinked shorter DNA fragments are preferred over a smaller number of larger fragments for resolving short branches.

## Conclusion

As the speed of genome sequencing continues to accelerate, phylogenomics is becoming a growing field of evolutionary biology. The potential of phylogenomics to address fundamental evolutionary questions has yet to be realized with the accumulation of phylogenomic studies for diverse groups of organisms [31-33]. The successful resolution of the rice phylogeny demonstrates the power of phylogenomics in the reconstruction of rapid evolutionary diversification. This study also highlights that organismal genomes might be mosaics of conflicting genealogies because of rapid speciation and exemplifies that phylogenetic relationships of organisms that undergo explosive or rapid diversification can be reliably resolved with increasing amounts of data and improved analytical methodology. A fully resolved rice phylogeny lays a solid foundation for comparative and functional genomic studies of rice and its related species and genera. Combined with the availability of rice genome sequences [2,64] and the BAC libraries of *Oryza *species representing all rice genome types [7], this phylogenetic framework will play an important role in the studies of genome evolution, speciation and adaptation, and crop domestication.

## Materials and methods

### Sampling single-copy genes

We used the BGI-RIS Database [39] for gene screening. Similar to the strategy used by Yu *et al. *[64], we extracted the protein sequences with nr-KOME cDNA [65] evidence and then conducted extensive searches against the genomic sequences of indica rice (*93-11*) in all six reading frames using TBLASTN at E-values of 10^-7^. To ensure that single-copy genes were used in our analysis, we applied a stringent similarity criterion of 50% in our searches; that is, only protein-coding genes that have no counterpart over 50% similar to themselves in the rice genome were selected for further analyses. Excluding those sequences without syntenic counterparts in the japonica (*Nipponbare*) genome [2], we got a total of 943 genes as candidates for phylogenetic markers. Using coding sequences of these candidates, we performed BLAST searches against the GenBank database to obtain the gene counterparts from barley, maize, sorghum, wheat, or other species of Poaceae as targets for primer design. On this basis, we designed 162 pairs of primers for amplifying orthologous segments from *Oryza *species and the outgroup *Leersia tisserantti*. Finally, 118 genes were kept according to the following criteria: they were sampled randomly from all the 12 rice chromosomes; the amplifying length ranged from 0.5-2.0 kb with an intron length of 30-70% so that adequate information is available at different taxonomic levels; and clear and strong amplified fragments were obtained from the *Oryza *species and the outgroup. Moreover, we sequenced 24 additional genes that were single copies demonstrated by previous studies (Additional data file 2). All the 142 genes used in this study were mapped onto the chromosomes of indica rice (*93-11*) (Additional data file 1).

### Species sampling, amplification, and sequencing

We sampled six *Oryza *species, representing all six diploid genomes in the genus, and one *Leersia *species (*L. tisserantti*) as outgroup because *Leersia *is most closely related to *Oryza *[12,53]. Information on the materials used in this study is listed in Table 1. Primers for PCR of all 142 genes are listed in Additional data file 14. Missing or partial sequences of some genes were present in some species because of the amplifying difficulty (Table 1). However, missing data in our case did not impact the tree constructions no matter what methods were used because our data set contained sufficient information, consistent with previous computer simulation and empirical investigation [21,25,66].

PCR amplifications and purification of the products were performed by standard methods. Purified products were sequenced either directly or after cloning into pGEM T-easy vectors (Promega, Madison, WI, USA) if the direct sequencing failed. Sequencing was carried out on an ABI 3730 automated sequencer (Applied Biosystems, Foster City, CA, USA). All sequences obtained in this study have been deposited in the GenBank database (accession numbers EF577518 to EF578433, and EU503348 to EU503533; Additional data file 14).

### Phylogenetic reconstructions

Individual genes were aligned using T-Coffee [67] and then manually adjusted. Phylogenetic trees were reconstructed by ML, MP and BI methods. ML and MP were implemented with PAUP 4.0b10 [68] and the branch-and-bound algorithm was used for tree searching. A non-parametric bootstrap strategy [69] was used for assessing tree reliability, with 1,000 replicates for MP analysis and with 100 and 500 replicates for ML analysis of the concatenated sequence and single genes, respectively.

BI was attempted with MrBayes 3.1.2 [70]. Given the sensitivity of the Bayesian method to model misspecification, we explored a series of homogeneous models by combining model components in different ways, including substitution rates among nucleotides (Nst = 1, 2, 6), rate variations across sites (Rates = Equal, Gamma, Propinv, Invgamma), and rate variations across the tree (Covarion = Yes, No) (Additional data file 6). Furthermore, we explored mixed models that accommodate heterogeneity across data partitions by specifying partition-specific substitution models [70]. We applied mixed models to our partitioned data by two schemes (see 'Analysis of systematic bias and congruence tests' below). Mixed models were implemented with separate models for each data partition selected by the program Modeltest 3.7 [71] and model parameters separately estimated, and a rate multiplier (ratepr = variable) was also employed to allow the overall rate to be different across partitions. In all the BI analyses, three independent Markov Chain Monte Carlo runs were executed, each starting with randomly choosing topologies for the four simultaneous chains, one cold and three incrementally heated. The four chains were run for at least 1,000,000 generations until stationarity in Markov chains was achieved, sampling trees every 100 generations with the first 10% of trees sampled discarded as burn-in, and then the posterior probabilities were calculated from the remaining samples.

We used Bayes factors [72] to evaluate the relative merits of two competing models, with the intention of detecting the effect of model components on our data. This method does not require alternative models to be hierarchically nested, and so it makes possible the comparison of any pair of distinctly different models. A Bayes factor in favor of one model (model 1) over another model (model 0) was calculated as the ratio of their marginal likelihoods and the natural logarithm of marginal likelihood can be approximated by the harmonic mean of the likelihoods of Markov Chain Monte Carlo samples with MrBayes [73]. We calculated twice the natural logarithm of the Bayes factors for the competing model pairs, and interpreted the results according to the rule suggested by Kass and Ratery [72], which states that a result of 2 to 6 is 'positive' evidence in favor of model 1, a result of 6 to 10 is 'strong' evidence, and a result of >10 is 'very strong' evidence; conversely, a result of <0 provides evidence in favor of model 0.

### Phylogenetic network analysis

To combine evidence from different loci without losing the information on independent gene histories, which might be drowned out by suppressing them into a bifurcating tree, several phylogenetic network approaches have been proposed and proven to be useful alternatives when using multi-gene data sets [74-76]. Consensus network, which is applied to multiple trees with the same set of taxa, is one commonly used network approach and can display simultaneously the conflicting evolutionary hypotheses based on multiple loci in a network fashion [74,76]. Such conflict or uncertainty might arise from stochastic errors, systematic bias, or biological processes [75]. Therefore, phylogenetic networks provide a more inclusive approach than analysis of the concatenated data set because weak or conflicting signals are hidden when genes are concatenated before phylogenetic analysis [76].

In the consensus network, areas where all trees have compatible splits (that is, a split is a bipartition of the taxa) will be tree-like (that is, a single branch); in contrast, areas with incompatible splits will be represented by bands of parallel edges, thus forming a potentially hyper-dimensional graph. The degree of denseness of boxes in networks reflects the intensity of contradictory evidence for grouping certain taxa, and the length of an edge is determined by the weight assigned to it [74,75]. The phylogenetic networks can range from one extreme, a structure of high-dimensional hypercubes in the absence of any common phylogenetic patterns among gene trees, to the other extreme, a unique bifurcating tree in the absence of stochasticity associated with bifurcating evolutionary process [75]. By employing the threshold value, we can reduce the visual complexity of resulting graphs by using only the splits that occur in more than a given proportion of all trees.

In the present study, we constructed consensus networks from optimal ML trees for a 106-gene data set in which sequences of all six diploid genomes and the outgroup were available and included in our consensus network all splits that occurred above a threshold value ranging from 0.05-0.3. In our case, branch lengths were not considered when using optimal ML trees as source trees because we were only interested in the conflict between topologies of gene trees. Thus, edge lengths in the final network are proportional to the number of trees in which a particular split appears. Consensus network was performed by the method described by Holland [76], in which Python scripts (kindly offered by BR Holland) was first implemented to create Nexus files and then the resulting network was visualized by Spectronet [77].

### Analysis of systematic bias and congruence tests

Systematic errors such as compositional signal, rate signal and heterotachous signal might be reinforced as more and more data are considered [35]. We first tested the compositional bias resulting from the heterogeneity of nucleotide compositions among lineages by Chi-square test. The LogDet distance [36] was also used to account for compositional bias with the neighbor-joining method. Then Tajima's relative rate test [37] was employed with each pair of *Oryza *species, using *L. tisserantti *as outgroup, to test rate constancy. Sequence data were also analyzed under the RY-coding strategy (A and G = R, C and T = Y), which maintains only transversions and thus efficiently reduces saturations by excluding more frequently occurring transitions [31,38]. In addition, the effect of heterotachous signal was explored by implementing a covarion model in BI.

Substitutional saturation of the data set was evaluated by plotting observed pairwise distance (uncorrected P-distance) for transitions and transversions against the ML pairwise distances for each pair of taxa. Saturation plots were constructed for total, exon, intron and third codon positions, respectively. Second order polynomial regression lines were fitted to all saturation plots and if the slope of this regression line was zero or negative, the data were considered saturated [78].

The ILD test [79], a character-based test for homogeneity, was used to explore the difference in phylogenetic signal between data partitions. We partitioned the data set by two schemes: four process partitions including intron and each codon positions [80]; and 142 gene partitions along gene boundaries, which may reveal variation in allelic histories that the concatenated data might obscure [26,76]. Then, we performed three kinds of ILD tests for each type of partition: a test among all partitions simultaneously; a test between all possible pairwise partitions; and a test between single partitions and the rest of the data set combined.

### Amount of sequence and phylogenetic resolution

To explore the relationship between the number of genes or nucleotides in a sample and the probability to infer the species tree in our case, we drew random samples of different sizes from the original 142-gene data set without replacement and concatenated each sample for phylogenetic analyses. When sampling genes, we generated samples consisting of 20, 40, 60, ..., 120 genes each for 500 replicates. Similarly, samples with randomly sampled sites in a total length of 10, 20, 30, ...100 kb were generated each for 500 replicates. ML and MP methods were used to determine whether or not the sampling results were affected by reconstruction methods. The branch-and-bound search was used in both methods, with the General Time Reversible (GTR)+Γ model for ML. The proportion of trees (or clades) identical to that in Figure 1 was calculated as the probability that a correct phylogenetic hypothesis will be obtained at a specific data size [63].

## Abbreviations

BI, Bayesian inference; GTR, General Time Reversible; ILD, incongruence length difference; kb, kilo base pairs; LogDet, log-determinant; ML, maximum likelihood; MP, maximum parsimony; Mya, million years ago; PP, Bayesian posterior probability.

## Additional data files

The following additional data are available with the online version of this paper. Additional data file 1 is a figure showing the relative location on rice chromosomes of the 142 genes sampled in this study. Additional file 2 is a table listing the detailed information on each of 142 loci. Additional file 3 is a table listing the GC content variation among lineages and the result of Chi-square test for the concatenated data set. Additional file 4 is a table summarizing the Tajima's relative rate test for concatenated sequences using *Leersia *as outgroup, with estimates of the ratio of substitution rate between lineages. Additional file 5 includes figures showing the results of testing the effect of rate bias caused by the fast-evolving genes of the F-genome. Additional file 6 is a table summarizing 14 alternative models used in BI analyses. Additional file 7 is a table indicating the effect of model components on model fit judged by Bayes factor comparisons of competing models. Additional file 8 is a figure showing the consensus networks of a collection of 106 optimal ML trees from the 106 genes with the complete set of seven species, applying thresholds of 0.05, 0.1, 0.15, 0.2, 0.25 and 0.3, respectively. Additional file 9 is a table presenting the results of the ILD test for pairwise comparisons of process partitions. Additional file 10 is a table summarizing the number of genes that failed the ILD test with the target gene at *P *< 0.01 for total, intron, exon and the third codon sites, respectively, and the *P *value of the ILD test between the target gene and all the rest of genes. Additional file 11 is a table presenting the topologies of bootstrap 75% majority-rule consensus trees by different methods of analyses for each gene. Additional file 12 is a figure showing saturation analyses in the concatenated datasets of total, intron, exon, and third codon positions, respectively. Additional file 13 is a table summarizing the proportions of topology (or clades) identical to those shown in Figure 1 inferred from randomly sampled genes or sites in 500 replicates. Additional file 14 is a table listing the primers for PCR amplification and the GenBank accession numbers of the sequences of 142 loci sampled in this present study.

## Authors' contributions

SG, XHZ, and TS designed the study; XHZ, FMZ, LLZ, and SG performed the research; FMZ, JGZ, and JW contributed the gene screening; XHZ, FMZ, LT, SG, and TS analyzed the data; SG, TS, and XHZ interpreted the data and wrote the paper.

## Supplementary Material

Additional data file 1 The relative location on rice chromosomes of the 142 genes sampled in this study. Click here for file

Additional data file 2 Detailed information on each of 142 loci. Click here for file

Additional data file 3 GC content variation among lineages and the result of Chi-square test for the concatenated data set. Click here for file

Additional data file 4 Summary of the Tajima's relative rate test for concatenated sequences using *Leersia *as outgroup, with estimates of the ratio of substitution rate between lineages. Click here for file

Additional data file 5 Results of testing the effect of rate bias caused by the fast-evolving genes of the F-genome. Click here for file

Additional data file 6 The 14 alternative models used in BI analyses. Click here for file

Additional data file 7 The effect of model components on model fit judged by Bayes factor comparisons of competing models Click here for file

Additional data file 8 Consensus networks of a collection of 106 optimal ML trees from the 106 genes with the complete set of seven species, applying thresholds of 0.05, 0.1, 0.15, 0.2, 0.25 and 0.3, respectively. Click here for file

Additional data file 9 Results of the ILD test for pairwise comparisons of process partitions. Click here for file

Additional data file 10 The number of genes that failed the ILD test with the target gene at *P *< 0.01 for total, intron, exon and the third codon sites, respectively, and the *P *value of the ILD test between the target gene and all the rest of genes. Click here for file

Additional data file 11 Topologies of bootstrap 75% majority-rule consensus trees by different methods of analyses for each gene. Click here for file

Additional data file 12 Saturation analyses in the concatenated datasets of total, intron, exon, and third codon positions, respectively. Click here for file

Additional data file 13 Proportions of topology (or clades) identical to those shown in Figure 1 inferred from randomly sampled genes or sites in 500 replicates. Click here for file

Additional data file 14 Primers for PCR amplification and the GenBank accession numbers of the sequences of 142 loci sampled. Click here for file
