# Supplementary material for: Analysis of 142 genes resolves the rapid diversification of the rice genus
Source: Genome Biol. 2008 Mar 3;9(3):R49. doi: 10.1186/gb-2008-9-3-r49 (PMC2397501; doi:10.1186/gb-2008-9-3-r49)
Supplement: Additional data file 1 — The relative location on rice chromosomes of the 142 genes sampled in this study. [file gb-2008-9-3-r49-S1.pdf]

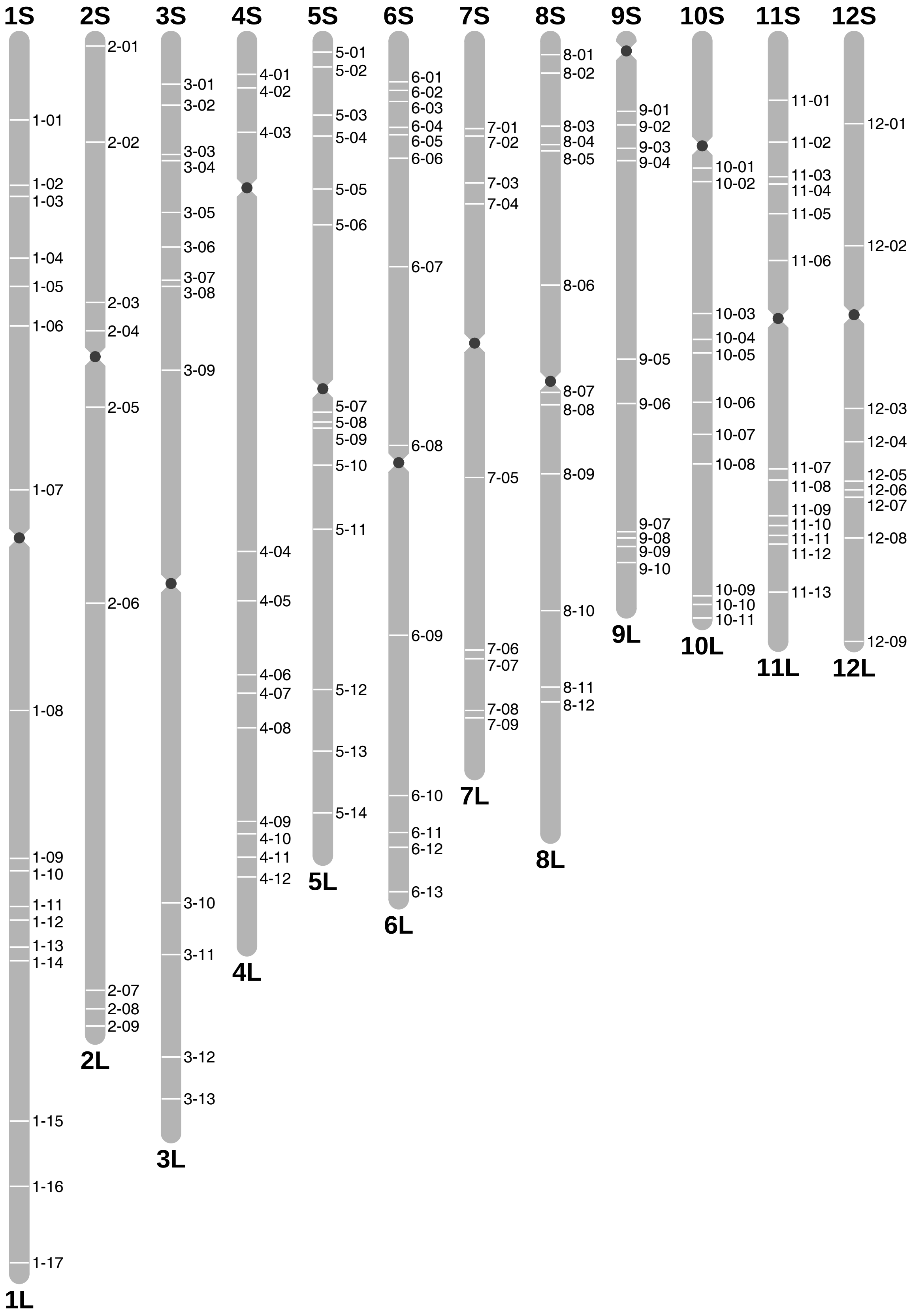
1S
1-01
1-02
1-03
1-04
1-05
1-06
1-07
1-08
1-09
1-10
1-11
1-12
1-13
1-14
1-15
1-16
1-17
1L
2S
2-01
2-02
2-03
2-04
2-05
2-06
2-07
2-08
2-09
2L
3S
3-01
3-02
3-03
3-04
3-05
3-06
3-07
3-08
3-09
3-10
3-11
3-12
3-13
3L
4S
4-01
4-02
4-03
4-04
4-05
4-06
4-07
4-08
4-09
4-10
4-11
4-12
4L
5S
5-01
5-02
5-03
5-04
5-05
5-06
5-07
5-08
5-09
5-10
5-11
5-12
5-13
5-14
5L
6S
6-01
6-02
6-03
6-04
6-05
6-06
6-07
6-08
6-09
6-10
6-11
6-12
6-13
6L
7S
7-01
7-02
7-03
7-04
7-05
7-06
7-07
7-08
7-09
7L
8S
8-01
8-02
8-03
8-04
8-05
8-06
8-07
8-08
8-09
8-10
8-11
8-12
8L
9S
9-01
9-02
9-03
9-04
9-05
9-06
9-07
9-08
9-09
9-10
9L
10S
10-01
10-02
10-03
10-04
10-05
10-06
10-07
10-08
10-09
10-10
10-11
10L
11S
11-01
11-02
11-03
11-04
11-05
11-06
11-07
11-08
11-09
11-10
11-11
11-12
11-13
11L
12S
12-01
12-02
12-03
12-04
12-05
12-06
12-07
12-08
12-09
12L

Additional data file 1. Relative location on rice chromosomes of the 142 genes sampled in this study. Numbers to the right of each chromosome are the gene ID used in this study. Numbers before the hyphen indicate the chromosome number and those after the hyphen represent the relative position of the genes in each chromosome (see Additional data file 2 for detailed information of the genes). S and L represent the short and long arms of the chromosomes, respectively.
