# Supplementary material for: Analysis of 142 genes resolves the rapid diversification of the rice genus
Source: Genome Biol. 2008 Mar 3;9(3):R49. doi: 10.1186/gb-2008-9-3-r49 (PMC2397501; doi:10.1186/gb-2008-9-3-r49)
Supplement: Additional data file 2 — Detailed information on each of 142 loci. [file gb-2008-9-3-r49-S2.pdf]

Additional data file 2. The detailed information of each of 142 loci sampled in the present study, including length of the alignment after excluding ambiguously regions (L), proportions of exon and GC content, ratio of transition and transversion (ti/tv), mean pairwise divergence (p-distance) (MPD) and percent of variable (VS) and parsimony informative sites (IS) among all taxa and within *Oryza* species, respectively. ID, gene identity defined by authors based on its location on rice chromosomes (see Additional data file 1); BGI and TIGR, gene identity in BGI-RIS and TIGR database, respectively.

ID	Taxa	L (bp)	Exon (%)	GC (%)	ti/tv	All taxa			*Oryza* species only			BGI	TIGR
						MPD (%)	VS (%)	IS (%)	MPD (%)	VS (%)	IS (%)		
1-01	ABCEFGL	810	24.81	40.11	1.71	9.79	26.79	5.43	8.81	21.85	4.20	OsIFCC000333 [1)]	LOC_Os01g05490.2
1-02	ABCEFGL	887	17.14	34.89	1.10	13.35	31.57	9.81	12.11	26.61	7.44	OsIFCC000631	LOC_Os01g09890.1
1-03	ABCEFGL	694	43.08	39.99	1.26	10.05	25.07	6.05	8.67	20.03	3.31	OsIFCC000666	LOC_Os01g10504.1
1-04	ABCEFGL	802	15.71	35.29	1.72	12.24	30.92	6.98	11.10	25.69	5.99	OsIFCC000930	LOC_Os01g13730.1
1-05	ABCEFGL	575	33.39	44.98	1.39	11.01	26.44	8.17	9.96	23.13	4.17	OsIFCC001048	LOC_Os01g15310.1
1-06	ABCEFGL	726	66.80	46.81	1.18	9.18	23.28	6.75	7.90	18.18	5.65	OsIFCC001201	LOC_Os01g17402.2
1-07	ABCEFGL	1081	56.52	39.78	1.90	9.24	22.02	6.01	8.03	18.13	4.63	OsIFCC001788	LOC_Os01g27730.1
1-08	ABCEL	699	54.51	41.67	1.21	8.23	17.45	2.43	5.34	9.44	1.00	OsIFCC002478	LOC_Os01g40670.2
1-09	ABCEFGL	999	66.47	40.62	1.12	6.69	18.12	3.70	5.86	14.11	3.00	OsIFCC003044 [2)]	LOC_Os01g48960.1
1-10	ABCEGL	923	18.20	40.01	1.61	11.63	25.46	7.04	9.90	19.72	4.01	OsIFCC003102	LOC_Os01g49690.1
1-11	ABCEF	919	19.70	40.48	1.52	9.75	20.35	1.63	9.75	20.35	1.63	OsIFCC003263	LOC_Os01g52110.9
1-12	ABCEFGL	682	48.83	38.50	1.36	8.31	21.55	5.28	6.87	16.28	3.96	OsIFCC003329	LOC_Os01g53020.1
1-13	ABCEL	988	31.28	40.88	1.69	7.67	17.92	1.22	5.13	9.82	0.91	OsIFCC003449	LOC_Os01g54910.1
1-14	ABCEFGL	954	43.08	40.11	1.59	7.52	20.23	4.09	6.81	16.77	3.15	OsIFCC003502 [3)]	LOC_Os01g55540.2
1-15	ABCEFGL	410	53.66	40.05	2.72	6.70	19.02	4.15	5.81	14.63	2.93	OsIFCC004159	LOC_Os01g65220.1
1-16	ABCEFGL	1565	72.65	47.74	2.41	8.05	21.34	6.01	7.16	17.38	4.47	OsIFCC004446 [4)]	LOC_Os01g69030.1
1-17	ABCEFGL	275	100.00	52.62	1.52	4.73	13.09	4.00	4.05	10.55	1.82	OsIFCC004786 [5)]	LOC_Os01g73890.1

2-01	ABCEFGL	704	44.46	39.76	1.23	11.59	29.69	6.96	10.67	25.57	5.54	OsIFCC012188	LOC_Os02g01350.1
2-02	ABCEFGL	736	44.43	37.99	1.51	8.92	23.64	5.57	8.05	19.57	3.53	OsIFCC012601	LOC_Os02g06584.1
2-03	ABCEFGL	804	22.01	36.06	1.24	12.22	30.97	8.58	11.03	25.37	6.34	OsIFCC013243	LOC_Os02g15900.1
2-04	ABCEFGL	1645	63.34	41.19	1.31	10.24	25.41	7.96	9.50	22.13	5.90	OsIFCC013341 [6)]	LOC_Os02g17780.1
2-05	ABCEFGL	1208	100.00	59.95	0.57	7.03	17.72	5.80	5.51	12.25	4.47	OsIFCC013615 [7)]	LOC_Os06g02019.1
2-06	ABCEFL	970	34.74	38.35	1.67	8.32	20.62	2.99	6.58	14.85	0.83	OsIFCC014205	LOC_Os02g33430.1
2-07	ABCEFGL	672	71.73	54.49	1.61	8.01	20.09	4.76	5.45	13.24	2.68	OsIFCC015762	LOC_Os02g56130.1
2-08	ABCEFGL	868	43.20	43.12	1.39	10.41	25.92	7.14	9.08	21.66	3.46	OsIFCC015825 [8)]	LOC_Os02g56850.2
2-09	ABCEFGL	778	34.96	44.22	0.88	10.29	26.22	6.94	8.91	21.08	4.76	OsIFCC015941	LOC_Os02g58220.1
3-01	ABCEFGL	1358	27.91	40.63	1.59	8.74	23.05	4.27	7.60	18.34	3.02	OsIFCC005013	LOC_Os03g03400.1
3-02	ABCEFL	891	24.92	38.76	1.42	10.36	24.02	3.93	8.68	17.62	1.46	OsIFCC005115	LOC_Os03g04460.1
3-03	ABCEFGL	515	45.24	41.01	1.76	7.62	20.97	4.66	6.27	15.15	3.69	OsIFCC005343	LOC_Os03g07790.1
3-04	ABCEFGL	1143	30.88	38.67	1.27	8.79	22.84	5.77	7.57	17.94	4.29	OsIFCC005351	LOC_Os03g07880.2
3-05	ABCEFGL	558	35.13	38.23	1.15	10.84	28.32	7.53	9.02	21.15	4.48	OsIFCC005617	LOC_Os03g11614.1
3-06	ABCEFGL	1243	38.29	39.48	1.75	7.85	20.43	4.83	6.96	16.73	3.70	OsIFCC005760	LOC_Os03g13614.1
3-07	ABCEFGL	639	71.05	47.20	1.68	8.68	22.69	5.79	6.94	15.96	4.54	OsIFCC005896	LOC_Os03g15700.1
3-08	ABCEFGL	823	28.31	36.95	1.37	10.41	26.12	6.80	9.30	20.90	5.10	OsIFCC005909	LOC_Os03g15860.1
3-09	ABCEFGL	964	51.14	41.11	1.70	6.51	17.64	3.73	5.08	12.34	2.91	OsIFCC006278	LOC_Os03g20100.1
3-10	ABCE	1318	58.27	46.63	1.12	4.36	8.04	0.38	4.36	8.04	0.38	OsIFCC008113	LOC_Os03g50540.1
3-11	ABCE	815	42.82	43.33	3.13	5.41	10.18	1.47	5.41	10.18	1.47	OsIFCC008323	LOC_Os03g53750.1
3-12	ABCEFGL	1129	36.94	39.60	1.19	10.42	26.40	7.71	8.98	20.99	4.96	OsIFCC008803	LOC_Os03g60939.1
3-13	ABCEFGL	753	47.94	37.65	1.53	7.53	18.46	7.70	7.58	18.46	4.38	OsIFCC009039	LOC_Os03g63940.1
4-01	ABCEFGL	624	81.89	48.65	1.66	4.84	12.66	4.33	4.31	10.42	2.72	OsIFCC016083	LOC_Os04g02900.1
4-02	ABCEFGL	882	64.06	39.03	1.35	6.72	19.27	3.40	5.58	14.63	2.15	OsIFCC016141	LOC_Os04g04020.1
4-03	ABCEFGL	976	27.97	35.72	1.66	9.19	24.59	5.02	8.21	19.57	4.00	OsIFCC016261 [9)]	LOC_Os04g08270.1
4-04	ABCEFGL	694	83.57	42.83	1.42	7.28	19.60	4.90	5.89	13.98	3.75	OsIFCC017591	LOC_Os04g35510.2
4-05	ABCEFGL	992	42.64	38.66	1.82	7.78	20.97	4.84	7.35	17.64	4.13	OsIFCC017814 [10)]	LOC_Os04g39020.1
4-06	ABCEFGL	675	41.33	39.91	1.49	9.07	23.11	5.19	8.04	18.67	3.70	OsIFCC018163	LOC_Os04g43300.1

4-07	ABCEFG	1191	20.57	35.98	1.10	9.82	22.84	5.04	9.82	22.84	5.04	OsIFCC018239	LOC_Os04g44430.1
4-08	ABCEFGL	738	65.58	39.75	1.98	8.16	21.95	5.42	6.87	17.07	3.39	OsIFCC018391	LOC_Os04g46450.1
4-09	ABCEFGL	1675	61.19	40.68	1.74	8.94	23.64	6.03	8.25	20.30	4.36	OsIFCC018763 11)	LOC_Os04g52230.1
4-10	ABCEFGL	1089	22.41	36.48	1.37	13.19	31.77	7.71	11.78	27.18	5.51	OsIFCC018831	LOC_Os04g52760.1
4-11	ABCEFGL	685	48.03	38.44	1.11	7.36	19.71	5.11	6.78	16.64	3.80	OsIFCC018940	LOC_Os04g54340.1
4-12	ABCEFGL	798	38.85	52.78	1.24	7.92	21.30	4.76	6.48	15.66	3.38	OsIFCC019034	LOC_Os04g55560.3
5-01	ABCEFGL	299	100.00	51.41	1.13	2.85	7.02	1.00	2.21	5.02	0.33	OsIFCC019404 12)	LOC_Os05g01710.3
5-02	ABCEFGL	731	57.73	38.34	1.81	6.72	19.29	3.69	6.19	15.87	2.74	OsIFCC019474	LOC_Os05g02670.1
5-03	ABCEFGL	867	23.53	39.34	1.58	10.38	26.07	6.69	9.10	20.65	4.84	OsIFCC019652	LOC_Os05g05260.1
5-04	ABCEFG	674	24.48	40.33	1.83	10.33	24.78	3.86	10.33	24.78	3.86	OsIFCC019756	LOC_Os05g06690.1
5-05	ABCEFGL	813	41.94	39.16	1.22	12.48	29.89	10.46	11.02	25.46	6.27	OsIFCC019936	LOC_Os05g09490.1
5-06	ABCEFGL	671	64.68	36.82	2.07	7.33	18.33	5.81	6.35	15.05	4.62	OsIFCC020049	LOC_Os05g11850.1
5-07	ABCEFGL	898	27.06	35.17	1.59	10.50	26.95	6.57	9.78	22.61	5.12	OsIFCC020589	LOC_Os05g23260.1
5-08	ABCEFGL	985	80.91	41.17	2.35	5.91	15.84	4.37	5.49	13.71	2.94	OsIFCC020611	LOC_Os05g23600.2
5-09	ABCEFGL	810	53.33	41.21	1.59	8.38	21.73	6.05	7.27	17.41	4.32	OsIFCC020613	LOC_Os05g23620.4
5-10	ABCEFGL	859	42.96	38.71	1.83	7.88	21.65	4.66	6.40	15.72	3.38	OsIFCC020758 13)	LOC_Os05g26890.4
5-11	ABCEFGL	264	100.00	48.70	1.60	10.48	23.49	14.39	7.99	20.46	2.65	OsIFCC020966	LOC_Os05g30530.1
5-12	ABCEF	1194	72.95	43.87	1.58	6.67	14.07	4.10	6.67	14.07	4.10	OsIFCC021563	LOC_Os05g39850.1
5-13	ACEFGL	951	34.70	39.77	1.34	9.18	21.98	5.15	8.34	16.72	3.79	OsIFCC021824	LOC_Os05g44310.1
5-14	ABCE	690	37.97	41.59	2.18	4.56	8.55	1.45	4.56	8.55	1.45	OsIFCC022111	LOC_Os05g49230.1
6-01	ABCFGL	567	33.33	38.89	1.36	11.12	25.93	7.41	10.03	20.64	6.00	OsIFCC009293	LOC_Os06g03600.2
6-02	ABCEFGL	1037	15.53	37.72	1.52	12.33	29.32	10.03	10.99	24.78	7.62	OsIFCC009322 14)	LOC_Os06g03990.1
6-03	ABCEFGL	732	88.52	57.84	1.78	6.05	14.34	6.83	4.88	10.66	5.06	OsIFCC009337 15)	LOC_Os06g04200.4
6-04	ABCEFGL	996	34.14	40.84	1.46	11.46	28.92	8.53	9.80	22.89	5.22	OsIFCC009509	LOC_Os06g06030.2
6-05	ABCEFGL	1294	26.12	37.48	1.42	10.31	25.35	6.49	8.84	19.86	4.56	OsIFCC009550 16)	LOC_Os06g06560.1
6-06	ABCEFGL	823	44.84	44.90	1.64	8.96	23.33	5.47	8.25	19.44	4.01	OsIFCC009655	LOC_Os06g08005.1
6-07	ABCEFGL	824	51.58	40.95	1.78	7.82	20.15	5.83	7.27	17.11	5.46	OsIFCC010070	LOC_Os06g13810.3
6-08	ABCEFGL	964	36.41	36.77	1.27	9.65	24.69	5.29	8.10	18.88	3.01	OsIFCC010608	LOC_Os06g24704.1

6-09	ABCEFGL	1079	100.00	52.10	1.53	9.43	26.32	4.54	5.86	14.64	2.87	OsIFCC011200 [17)]	LOC_Os06g37364.2
6-10	ABCEFGL	791	46.52	39.91	1.58	9.36	24.78	7.59	8.22	20.10	4.93	OsIFCC011734	LOC_Os06g45000.2
6-11	ABCEFGL	518	58.11	41.12	1.53	8.84	22.39	7.72	7.76	17.95	6.37	OsIFCC011884	LOC_Os06g46930.1
6-12	ABCEG	1289	50.04	42.71	1.78	3.65	7.68	2.33	3.65	7.68	2.33	OsIFCC011941	LOC_Os06g47840.1
6-13	ABCEFGL	742	49.19	42.98	1.72	8.41	22.91	4.99	6.98	16.98	2.83	OsIFCC012135 [18)]	LOC_Os06g51150.2
7-01	ABCEFG	1362	49.05	41.66	2.11	7.79	18.58	4.41	7.79	18.58	4.41	OsIFCC022658	LOC_Os07g07060.1
7-02	ABCEFGL	731	29.82	37.43	1.23	10.02	26.81	5.88	8.90	21.75	3.97	OsIFCC022675	LOC_Os07g07260.2
7-03	ABCF	529	14.37	40.96	2.21	8.23	15.12	0.38	8.23	15.12	0.38	OsIFCC022875	LOC_Os07g10130.1
7-04	ABCEF	1091	7.61	39.75	1.04	10.41	22.00	1.74	10.41	22.00	1.74	OsIFCC022941	LOC_Os07g11060.2
7-05	ABCEFGL	1054	60.25	42.89	1.17	7.84	21.63	4.36	7.01	17.55	2.85	OsIFCC023726	LOC_Os07g31340.2
7-06	ABCEFGL	1076	49.07	42.54	1.32	11.02	27.70	9.11	10.07	23.70	5.67	OsIFCC024396	LOC_Os07g42300.2
7-07	ABCEFGL	938	42.75	38.55	1.88	9.49	24.20	6.40	8.08	19.19	3.63	OsIFCC024429	LOC_Os07g42810.2
7-08	ABCEFGL	993	19.64	38.32	2.03	11.50	25.18	5.94	10.51	21.45	4.83	OsIFCC024668	LOC_Os07g46440.2
7-09	ABCEFGL	457	50.33	41.82	1.28	10.34	25.82	8.97	9.21	21.23	7.00	OsIFCC024700	LOC_Os07g46750.1
8-01	ABCEFGL	575	37.91	38.41	1.65	9.11	23.48	6.61	8.11	19.13	3.83	OsIFCC024974	LOC_Os08g01740.2
8-02	ABCFL	1246	43.10	48.02	1.17	10.42	20.55	5.62	9.35	16.53	1.85	OsIFCC025045	LOC_Os08g02640.1
8-03	ABCEFGL	691	50.07	44.72	2.00	8.75	21.42	7.96	7.78	17.66	5.93	OsIFCC025228	LOC_Os08g05830.1
8-04	ABCEFGL	693	41.99	43.33	1.74	8.58	20.64	8.23	8.66	20.64	4.19	OsIFCC025278	LOC_Os08g06530.1
8-05	ABCEFGL	575	38.61	41.20	1.51	9.13	23.65	5.39	8.35	20.00	4.17	OsIFCC025286	LOC_Os08g06630.4
8-06	ABCG	735	68.30	49.13	1.59	3.67	6.53	1.77	3.67	6.53	1.77	OsIFCC025775	LOC_Os08g14450.1
8-07	ABCEFG	2082	57.78	40.80	1.44	5.36	13.55	2.69	5.36	13.55	2.69	OsIFCC026144 [19)]	LOC_Os08g20660.2
8-08	ABCEFGL	634	35.80	35.41	1.60	8.59	22.87	5.99	6.95	16.56	4.26	OsIFCC026184 [20)]	LOC_Os08g22354.1
8-09	ABCFGL	590	15.76	39.96	1.08	14.23	31.02	8.81	11.77	22.54	6.44	OsIFCC026346	LOC_Os08g25460.1
8-10	ABCEFGL	666	33.48	37.26	1.85	10.66	28.38	7.06	9.23	22.07	5.56	OsIFCC026796	LOC_Os08g33076.1
8-11	ABCEFGL	772	72.67	56.06	1.24	7.94	21.76	4.40	7.32	18.14	3.37	OsIFCC027043	LOC_Os08g36790.1
8-12	ABCEFGL	888	53.72	41.17	1.19	9.09	24.66	5.18	8.34	20.72	3.38	OsIFCC027081	LOC_Os08g37444.5
9-01	ABCEFGL	460	41.96	37.99	0.98	7.85	20.00	6.09	6.68	15.65	4.57	OsIFCC027859	LOC_Os09g06499.1
9-02	ABCEFGL	1238	66.64	45.52	2.38	6.21	17.12	3.31	5.05	12.84	2.18	OsIFCC027894	LOC_Os09g07300.1

9-03	ABCFGL	1210	22.73	36.80	1.17	10.66	24.55	4.71	9.77	19.50	3.64	OsIFCC027959	LOC_Os09g08390.4
9-04	ABCEFGL	590	51.53	36.42	1.61	8.58	22.20	7.29	7.89	18.31	6.10	OsIFCC027996	LOC_Os09g09480.2
9-05	ABCEFGL	859	63.56	40.73	1.76	7.49	20.49	5.47	7.12	17.11	4.77	OsIFCC028658	LOC_Os09g22450.1
9-06	ABCEGL	1230	17.40	36.87	1.07	10.84	22.93	5.45	9.38	16.26	3.09	OsIFCC028803	LOC_Os09g25640.1
9-07	ABCEFGL	1307	35.04	37.33	1.71	8.56	22.88	5.51	7.61	18.75	4.21	OsIFCC029321	LOC_Os09g33980.1
9-08	ABCEFGL	793	61.54	42.04	1.67	8.11	20.43	7.31	6.96	16.27	5.68	OsIFCC029322	LOC_Os09g33986.2
9-09	ABCEFGL	877	64.20	39.82	1.68	7.63	20.07	5.82	7.09	17.10	3.99	OsIFCC029340	LOC_Os09g34190.1
9-10	ABCEFGL	443	61.17	49.82	1.25	7.18	17.38	6.77	6.31	14.45	4.06	OsIFCC029466	LOC_Os09g36800.1
10-01	ABCEFGL	637	29.20	37.21	2.01	8.23	20.72	5.18	7.21	17.27	2.67	OsIFCC030113	LOC_Os10g10190.1
10-02	ABCE	2086	19.13	42.11	1.37	5.65	10.07	1.92	5.65	10.07	1.92	OsIFCC030157	LOC_Os10g11140.1
10-03	ABCE	754	23.87	42.74	2.13	4.68	8.62	0.27	4.68	8.62	0.27	OsIFCC030576	LOC_Os10g23120.1
10-04	ABCEGL	372	51.61	39.18	0.94	6.51	16.67	2.15	4.54	10.22	1.08	OsIFCC030673	LOC_Os10g25320.3
10-05	ABCEFG	1023	20.82	42.46	1.34	11.59	26.00	5.96	11.59	26.00	5.96	OsIFCC030724	LOC_Os10g26140.2
10-06	ABCEFGL	748	43.58	40.72	1.26	7.84	19.92	6.15	6.90	16.18	3.61	OsIFCC030908	LOC_Os10g29660.1
10-07	ABCEFGL	996	31.02	37.10	1.28	9.24	24.80	5.02	7.60	18.57	3.82	OsIFCC031025	LOC_Os10g31520.2
10-08	ABCEFGL	453	25.83	37.08	1.21	11.89	31.57	6.40	10.48	24.72	4.64	OsIFCC031142	LOC_Os10g33270.1
10-09	ABCEL	739	21.11	40.30	1.60	5.69	13.26	0.41	2.39	4.74	0.14	OsIFCC031711	LOC_Os10g41340.1
10-10	ABCE	876	65.64	44.79	2.05	2.65	4.91	0.91	2.65	4.91	0.91	OsIFCC031753	LOC_Os10g41960.1
10-11	ABCEFGL	603	0.00	38.23	1.55	12.23	30.85	6.80	10.48	24.38	5.31	OsIFCC031807 [21)]	LOC_Os10g42720.1
11-01	ABCEGL	1158	33.07	40.00	1.60	8.15	19.52	4.49	6.55	14.51	1.21	OsIFCC034196	---[*]
11-02	ABCEFGL	843	41.16	38.38	1.34	11.56	25.86	10.08	10.60	22.54	8.54	OsIFCC034358	LOC_Os11g07916.1
11-03	ABCEFGL	937	63.39	45.81	1.56	8.11	20.60	7.47	7.25	16.65	5.55	OsIFCC034489 [22)]	LOC_Os11g10480.2
11-04	ABCEFGL	1109	53.56	47.93	1.21	8.94	22.54	7.48	8.22	19.30	5.86	OsIFCC034491 [23)]	LOC_Os11g10510.1
11-05	ABCEFGL	970	14.43	33.63	1.25	14.83	31.34	11.75	12.92	24.85	10.41	OsIFCC034620	LOC_Os11g13810.1
11-06	ABCEFG	689	18.72	37.78	1.21	11.85	26.27	6.82	11.85	26.27	6.82	OsIFCC034777	LOC_Os11g17610.1
11-07	ABCEFGL	914	33.15	36.20	1.32	11.54	29.21	9.08	9.09	21.77	5.80	OsIFCC035427	LOC_Os11g33090.1
11-08	ABCEFGL	844	15.76	34.69	1.43	9.51	23.58	7.82	8.99	19.79	6.64	OsIFCC035472	LOC_Os11g34200.2
11-09	ABCE	1098	24.32	41.95	1.52	8.62	14.85	4.28	8.62	14.85	4.28	OsIFCC035610	LOC_Os11g36390.1

11-10	ABCEFGL	639	32.39	40.87	2.08	9.64	25.98	5.79	8.69	21.13	3.91	OsIFCC035654	LOC_Os11g37130.1
11-11	ABCEFGL	918	27.12	36.02	1.47	8.82	23.42	5.45	8.48	20.48	4.68	OsIFCC035694	LOC_Os11g38990.3
11-12	ABCEFGL	964	6.02	36.06	1.36	14.55	31.02	8.40	12.69	24.79	6.85	OsIFCC035700	LOC_Os11g38920.1
11-13	ABCEFGL	386	70.47	43.49	2.24	6.32	16.58	5.18	5.83	14.25	3.63	OsIFCC035910	LOC_Os11g43510.1
12-01	ABCEFGL	889	37.57	35.60	1.70	9.18	24.30	5.96	8.04	19.46	4.27	OsIFCC032170	LOC_Os12g06200.2
12-02	ABCEFGL	1088	51.01	39.27	1.36	11.46	28.40	9.74	10.44	23.90	7.17	OsIFCC032605 [24)]	LOC_Os12g15470.1
12-03	ABCFGL	631	17.27	38.53	1.23	11.78	25.04	5.07	11.05	21.24	3.49	OsIFCC033021	LOC_Os12g29660.1
12-04	ABCEFGL	683	38.80	36.12	1.58	9.35	24.31	5.86	7.81	18.74	3.66	OsIFCC033140	LOC_Os12g31810.1
12-05	ABCEFGL	787	63.91	38.05	1.56	6.75	17.92	4.57	6.19	15.12	2.92	OsIFCC033292	LOC_Os01g27750.1
12-06	ABCEFGL	777	30.63	35.68	1.55	10.80	27.80	6.18	10.20	23.81	5.41	OsIFCC033332	LOC_Os12g35570.3
12-07	ABCEGL	478	69.87	48.60	1.74	4.82	12.13	2.93	3.97	9.21	1.05	OsIFCC033336	LOC_Os12g35630.1
12-08	ABCEFGL	1206	30.85	36.99	1.43	9.67	24.46	6.55	8.68	20.32	4.73	OsIFCC033507	LOC_Os12g38380.1
12-09	ABCE	1495	31.91	42.86	1.23	5.07	9.70	0.20	5.07	9.70	0.20	OsIFCC033909	---
Total	ABCEFGL	124079	43.10	41.07	1.40	9.22	21.63	5.44	8.13	17.76	3.95	---	---

[*] not available

[1) ~ 24)] stand for the 24 genes that were single copies demonstrated by the following studies:

1) *TPI* [1]; 2) *NADH-GOGAT* [2]; 3) *AAT1* [3]; 4) *SPS* [4]; 5) *TFIIAγ1* [5]; 6) *CPS1* [6]; 7) *KAO* [6]; 8) *RGRC2* [7]; 9) *SDBE* [8]; 10) *BADH* [9]; 11) *KS1* [6]; 12) *TFIIAγ5* [5]; 13) *GPA1* [10]; 14) *ACS2* [11]; 15) *Waxy* [12]; 16) *SSS1* [13]; 17) *KO2* [6]; 18) *CatB* [14]; 19) *CEN6733* [15]; 20) *CEN6729* [15]; 21) *GPAT* [16]; 22) *Adh1* [17]; 23) *Adh2* [17]; 24) *CBP1* [18].

References

1. Xu Y, Hall TC: **Cytosolic triosephosphate isomerase is a single gene in rice**. *Plant Physiol* 1993, **101**:683-687.
2. Goto S, Akagawa T, Kojima S, Hayakawa T, Yamaya T: **Organization and structure of NADH-dependent glutamate synthase**

gene from rice plants. *Biochimica et biophysica acta* 1998, **1387**:298-308.
3. Song J, Yamamoto K, Shomura A, Yano M, Minobe Y, Sasaki T: **Characterization and mapping of cDNA encoding aspartate aminotransferase in rice, *Oryza sativa* L.** *DNA Res* 1996, **3**:303-310.
4. Sakamoto M, Satozawa T, Kishimoto N, Higo K, Shimada H, Fujimura T: **Structure and RFLP mapping of a rice sucrose phosphate synthase (SPS) gene that is specifically expressed in the source organ**. *Plant Sci* 1995, **112**:207-217.
5. Blair MW, Garris AJ, Iyer AS, Chapman B, Kresovich S, McCouch SR: **High resolution genetic mapping and candidate gene identification at the xa5 locus for bacterial blight resistance in rice (*Oryza sativa* L.)**. *Theor Appl Genet* 2003, **107**:62-73.
6. Sakamoto T, Miura K, Itoh H, Tatsumi T, Ueguchi-Tanaka M, Ishiyama K, Kobayashi M, Agrawal GK, Takeda S, Abe K *et al.*: **An overview of gibberellin metabolism enzyme genes and their related mutants in rice**. *Plant Physiol* 2004, **134**:1642-1653.
7. Kaminaka H, Morita S, Nakajima M, Masumura T, Tanaka K: **Gene cloning and expression of cytosolic glutathione reductase in rice (*Oryza sativa* L.)**. *Plant Cell Physiol* 1998, **39**:1269-1280.
8. Nakamura Y, Umemoto T, Ogata N, Kuboki Y, Yano M, Sasaki T: **Starch debranching enzyme (R-enzyme or pullulanase) from developing rice endosperm: purification, cDNA and chromosomal localization of the gene**. *Planta* 1996, **199**:209-218.
9. Nakamura T, Yokota S, Muramoto Y, Tsutsui K, Oguri Y, Fukui K, Takabe T: **Expression of a betaine aldehyde dehydrogenase gene in rice, a glycinebetaine nonaccumulator, and possible localization of its protein in peroxisomes**. *Plant J* 1997, **11**:1115-1120.
10. Seo HS, Kim HY, Jeong JY, Lee SY, Cho MJ, Bahk JD: **Molecular cloning and characterization of *RGA1* encoding a G protein alpha subunit from rice (*Oryza sativa* L. IR-36)**. *Plant Mol Biol* 1995, **27**:1119-1131.
11. Zarembinski TI, Theologis A: **Expression characteristics of *OS-ACS1* and *OS-ACS2*, two members of the 1-aminocyclopropane-1-carboxylate synthase gene family in rice (*Oryza sativa* L. cv. Habiganj Aman II) during partial submergence**. *Plant Mol Biol* 1997, **33**:71-77.
12. Dian W, Jiang H, Chen Q, Liu F, Wu P: **Cloning and characterization of the granule-bound starch synthase II gene in rice: gene expression is regulated by the nitrogen level, sugar and circadian rhythm**. *Planta* 2003, **218**:261-268.
13. Baba T, Nishihara M, Mizuno K, Kawasaki T, Shimada H, Kobayashi E, Ohnishi S, Tanaka K, Arai Y: **Identification, cDNA cloning, and gene expression of soluble starch synthase in rice (*Oryza sativa* L.) immature seeds**. *Plant Physiol* 1993, **103**:565-573.
14. Iwamoto M, Maekawa M, Saito A, Higo H, Higo K: **Evolutionary relationship of plant catalase genes inferred from exon-intron structures: isozyme divergence after the separation of monocots and dicots**. *Theor Appl Genet* 1998, **97**:9-19.

15. Nagaki K, Cheng Z, Ouyang S, Talbert PB, Kim M, Jones KM, Henikoff S, Buell CR, Jiang J: **Sequencing of a rice centromere uncovers active genes**. *Nat Genet* 2004, **36**:138-145.
16. Yokoi S, Higashi S, Kishitani S, Murata N, Toriyama K: **Introduction of the cDNA for *Arabidopsis* glycerol-3-phosphate acyltransferase (GPAT) confers unsaturation of fatty acids and chilling tolerance of photosynthesis on rice**. *Mol Breed* 1998, **4**:269-275.
17. Xie Y, Wu R: **Rice alcohol dehydrogenase genes: anaerobic induction, organ specific expression and characterization of cDNA clones**. *Plant Mol Biol* 1989, **13**:53-68.
18. Washio K, Ishikawa K: **Organ-specific and hormone-dependent expression of genes for serine carboxypeptidases during development and following germination of rice grains**. *Plant Physiol* 1994, **105**:1275-1280.
