# Supplementary material for: Analysis of 142 genes resolves the rapid diversification of the rice genus
Source: Genome Biol. 2008 Mar 3;9(3):R49. doi: 10.1186/gb-2008-9-3-r49 (PMC2397501; doi:10.1186/gb-2008-9-3-r49)
Supplement: Additional data file 3 — GC content variation among lineages and the result of Chi-square test for the concatenated data set. [file gb-2008-9-3-r49-S3.pdf]

Additional data file 3. GC content variation among lineages and Chi-square test for the concatenated data set. The capital letters under GC (%) indicate the genome types except that L stands for *Leersia tisserantti*.

Data set	No. sites (bp)	GC (%)								
		A	B	C	E	F	G	L	Mean	P *
All sites	124,079	40.1	40.1	40.1	40.1	40.6	40.5	40.2	40.2	0.6766
Intron	70,600	34.6	34.6	34.6	34.7	34.6	34.8	34.5	34.7	0.9998
Exon	53,479	46.9	46.9	46.9	46.7	47.4	47.0	46.6	46.9	0.9420
1st codon	17,828	55.0	55.0	54.9	54.9	55.0	55.0	54.6	54.9	0.9998
2nd codon	17,827	39.8	39.7	39.7	39.8	39.8	39.7	39.9	39.8	1.0000
3rd codon	17,824	45.8	45.7	46.0	45.4	47.3	46.2	45.4	46.0	0.3463
Intron+3rd codon	88,424	37.0	37.0	37.1	37.0	37.5	37.4	37.0	37.1	0.8376

* Pairwise χ^2 tests.
