# Supplementary material for: Analysis of 142 genes resolves the rapid diversification of the rice genus
Source: Genome Biol. 2008 Mar 3;9(3):R49. doi: 10.1186/gb-2008-9-3-r49 (PMC2397501; doi:10.1186/gb-2008-9-3-r49)
Supplement: Additional data file 4 — Summary of the Tajima's relative rate test for concatenated sequences using Leersia as outgroup, with estimates of the ratio of substitution rate between lineages. [file gb-2008-9-3-r49-S4.pdf]

Additional data file 4. Results of Tajima's relative rate test for concatenated sequences using *Leersia* as outgroup, with estimates of the ratio of substitution rate between lineages. Numbers in each cell is the ratio (r) of substitution rate of row over column, and an r >1 means that the row is estimated to have faster substitution rate than the column (Bold face). For example, F-genome is estimated to evolve significantly faster than all other genomes at all datasets. The asterisk denotes the violation of hypothesis of rate constancy by relative rate test. Letters (A to G) indicate the genome types.

**Genome**	**A**	**B**	**C**	**E**	**F**
**B**	0.87*				
**C**	0.79*	0.90*			
**E**	0.86*	0.96	**1.04**		
**F**	**1.30***	**1.38***	**1.44***	**1.41***	
**G**	0.70*	0.75*	0.78*	0.76*	0.54*

* $P < 0.01$.

Note. The ratio of substitution rate between two lineages is calculated by method modified from Gaut et al. [1], which is given by

$$r_{12} = \frac{d_{10} + d_{12} - d_{20}}{d_{20} + d_{12} - d_{10}},$$

, where d_{10} is the distance (Kimura 2-parameter distance) between lineage 1 and the outgroup, d_{20} is the distance between lineage 2 and the outgroup, and d_{12} is the distance between lineage 1 and lineage 2.

References

1. Gaut BS, Clark LG, Wendel JF, Muse SV: **Comparisons of the molecular**

evolutionary process at *rbcL* and *ndhF* in the grass family (Poaceae). *Mol Biol Evol* 1997, **14**:769-777.
