# Supplementary material for: Analysis of 142 genes resolves the rapid diversification of the rice genus
Source: Genome Biol. 2008 Mar 3;9(3):R49. doi: 10.1186/gb-2008-9-3-r49 (PMC2397501; doi:10.1186/gb-2008-9-3-r49)
Supplement: Additional data file 5 — Results of testing the effect of rate bias caused by the fast-evolving genes of the F-genome. [file gb-2008-9-3-r49-S5.pdf]

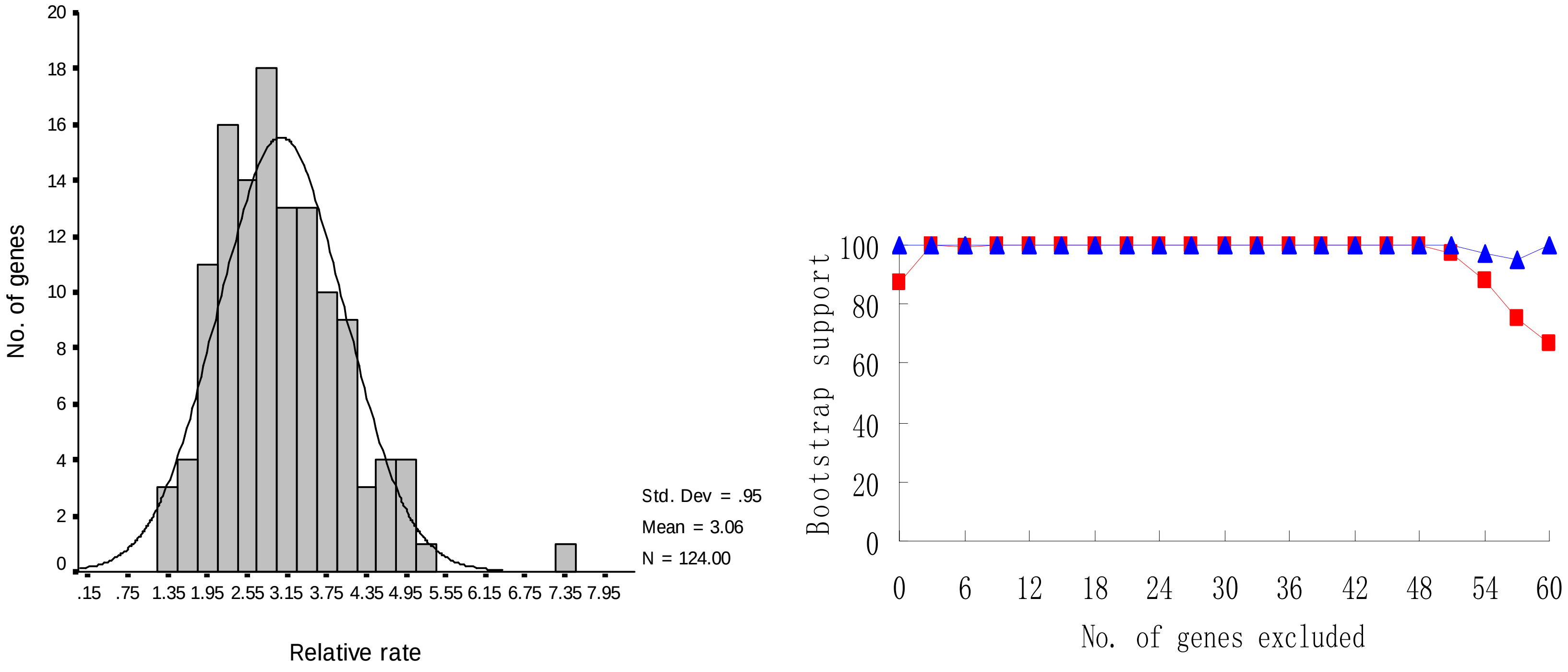

Additional data file 5. Testing the effect of rate bias caused by the fast-evolving genes of the F-genome. Left: Histogram of relative evolutionary rates of the F-genome for 142 genes, which is represented by the ratio of the mean distance between the F-genome and each of the A-, B-, and C-genomes to the average of the distances among the A-, B-, and C-genomes for each gene. Right: Bootstrap support for placing the G-genome at the basal position when sequentially and cumulatively discarding the fast-evolving genes of the F-genome in decreasing order three at a time and reconstructed the phylogeny for each pruned data set. The blue triangles represent the results of ML method and the red squares the results of MP method.
