# Supplementary material for: Analysis of 142 genes resolves the rapid diversification of the rice genus
Source: Genome Biol. 2008 Mar 3;9(3):R49. doi: 10.1186/gb-2008-9-3-r49 (PMC2397501; doi:10.1186/gb-2008-9-3-r49)
Supplement: Additional data file 6 — The 14 alternative models used in BI analyses. [file gb-2008-9-3-r49-S6.pdf]

Additional data file 6. Summary of 14 alternative models used when Bayesian inferences were conducted. The models contained 11 homogeneous models and three mixed models in which parameters were unlinked across partitions. The number of active parameters and harmonic means of model likelihood were listed. JC = Jukes-Cantor model; HKY = Hasegawa-Kishino-Yano model; GTR = general time reversible model; I = proportion of invariant sites; Γ = gamma rate distribution; covarion = covarion-like model in Mrbayes; 4 process partition = intron + 1st codon position + 2nd codon position + 3rd codon position; 2 process partition = intron + exon; 142 gene partition = partitions by gene boundaries.

Model category	Model	Harmonic mean (in decreasing order)	No. of active parameters
mixed models	4 process partition	-328024.03	16
mixed models	2 process partition	-332339.84	10
mixed models	142 gene partition	-336533.69	410
homogeneous model	GTR+covarion	-337703.76	5
homogeneous model	HKY+covarion	-337797.42	5
homogeneous model	GTR+Γ+I	-337808.09	6
homogeneous model	GTR+Γ	-337808.23	5
homogeneous model	GTR+I	-337832.36	5
homogeneous model	HKY+Γ	-337903.95	5
homogeneous model	HKY+Γ+I	-337905.39	6
homogeneous model	HKY+I	-337922.85	5
homogeneous model	GTR	-339606.91	4
homogeneous model	HKY	-339717.15	4
homogeneous model	JC	-344589.77	3
