# Supplementary material for: Analysis of 142 genes resolves the rapid diversification of the rice genus
Source: Genome Biol. 2008 Mar 3;9(3):R49. doi: 10.1186/gb-2008-9-3-r49 (PMC2397501; doi:10.1186/gb-2008-9-3-r49)
Supplement: Additional data file 7 — The effect of model components on model fit judged by Bayes factor comparisons of competing models [file gb-2008-9-3-r49-S7.pdf]

Additional data file 7. Effect of model components on model fit judged by Bayes factor comparisons of competing models.

Model component	Paired models for comparison		Harmonic means of models		Bayes factor	
	Model_0	Model_1	Model_0	Model_1	$\log_e B_{10}$	$2\log_e B_{10}$
Rate variation across sites	HKY	HKY+Γ	-339717.15	-337903.95	1813.20	3626.40
Rate variation across sites	GTR	GTR+Γ	-339606.91	-337808.23	1798.68	3597.36
Rate variation across sites	HKY	HKY+I	-339717.15	-337922.85	1794.30	3588.60
Rate variation across sites	GTR	GTR+I	-339606.91	-337832.36	1774.55	3549.10
Rate variation across the tree	HKY	HKY+covarion	-339717.15	-337797.42	1919.73	3839.46
Rate variation across the tree	GTR	GTR+covarion	-339606.91	-337703.76	1903.15	3806.30
Substitution rates	HKY	GTR	-339717.15	-339606.91	110.24	220.48
Substitution rates	HKY+Γ	GTR+Γ	-337903.95	-337808.23	95.72	191.44
Substitution rates	HKY+Γ+I	GTR+Γ+I	-337905.39	-337808.09	97.30	194.60
Rate variation across sites	HKY+Γ	HKY+Γ+I	-337903.95	-337905.39	-1.44	-2.88
Rate variation across sites	GTR+Γ	GTR+Γ+I	-337808.23	-337808.09	0.14	0.28
Rate variation across sites	HKY+I	HKY+Γ+I	-337922.85	-337905.39	17.46	34.92
Rate variation across sites	GTR+I	GTR+Γ+I	-337832.36	-337808.09	24.27	48.54
Data partition	2 process partition	4 process partition	-332339.84	-328024.03	4315.81	8631.62
Data partition	142 gene partition	2 process partition	-336533.69	-332339.84	4193.85	8387.70
Data partition	GTR+covarion	142 gene partition	-337703.76	-336533.69	1170.07	2340.14

Data partition	GTR+covarion	4 process partition	-337703.76	-328024.03	9679.73	19359.46

Notes: Bayes factor in favor of model_1 over model_0 is denoted by B_{10}. Bayes factor comparisons indicated that among most of competing model pairs, model_1 got a significantly great improvement in model fit compared to model_0. For homogeneous models, models assuming rates varying across sites or across the tree got extraordinarily high supports by over 3500 log likelihood units relative to corresponding ones with equal rates, and allowing nucleotide substitution under GTR model was favoured over HKY model with about 200 log likelihood units, which is also far above the threshold of 10 log likelihood units for the very strong evidence in favour of the better model [1]. For mixed models, those accounting for the heterogeneity across two process partitions outperformed that accounting for the heterogeneity across 142 gene partitions by 8387.7 log likelihood units, and a dramatic increase of 8631.62 log likelihood units was obtained when partitioning the data from 2 partitions into 4 partitions by functional constrains.

References

1. Kass RE, Raftery AE: **Bayes factors**. *J Am Stat Assoc* 1995, **90**:773-795.
