# Supplementary material for: Analysis of 142 genes resolves the rapid diversification of the rice genus
Source: Genome Biol. 2008 Mar 3;9(3):R49. doi: 10.1186/gb-2008-9-3-r49 (PMC2397501; doi:10.1186/gb-2008-9-3-r49)
Supplement: Additional data file 8 — Consensus networks of a collection of 106 optimal ML trees from the 106 genes with the complete set of seven species, applying thresholds of 0.05, 0.1, 0.15, 0.2, 0.25 and 0.3, respectively. [file gb-2008-9-3-r49-S8.pdf]

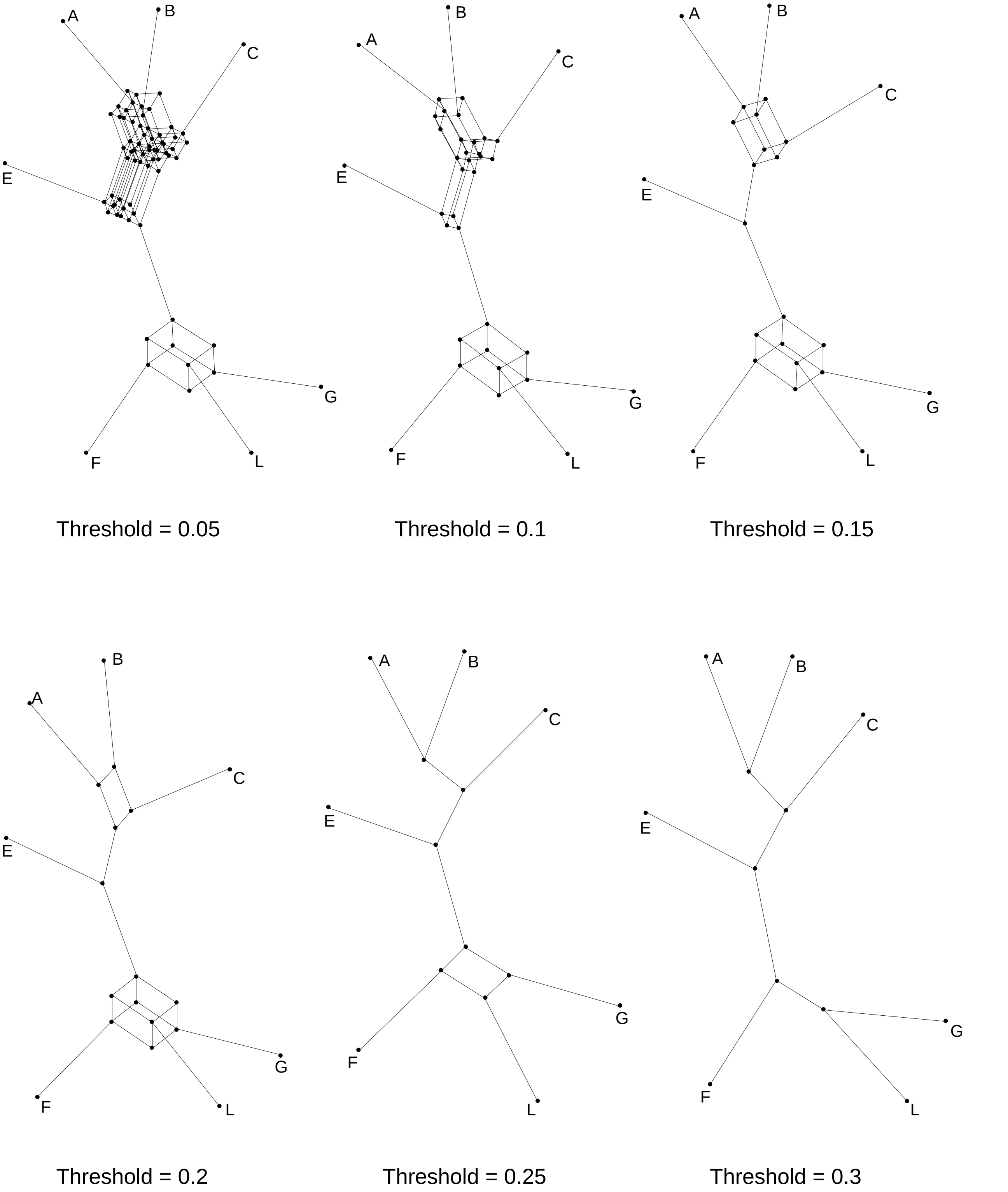

Additional data file 8. Consensus networks of collection of 106 optimal ML trees from the 106 genes with complete set of seven species applying the threshold of 0.05, 0.1, 0.15, 0.2, 0.25 and 0.3, respectively. Letters (A to L) are the same to those in figure 1.
