# Supplementary material for: Analysis of 142 genes resolves the rapid diversification of the rice genus
Source: Genome Biol. 2008 Mar 3;9(3):R49. doi: 10.1186/gb-2008-9-3-r49 (PMC2397501; doi:10.1186/gb-2008-9-3-r49)
Supplement: Additional data file 9 — Results of the ILD test for pairwise comparisons of process partitions. [file gb-2008-9-3-r49-S9.pdf]

Additional data file 9. Results of incongruence length difference (ILD) test for pairwise comparisons of process partitions. Numbers in each cell is the *P* value of ILD test between row and column.

**Partition**	1st codon	2nd codon	3rd codon	Intron	Exon
2nd codon	0.273				
3rd codon	0.203	0.171			
Intron	0.062	0.102	0.999		
All the rest	0.057	0.140	0.999	0.109	0.109
