# Supplementary material for: Analysis of 142 genes resolves the rapid diversification of the rice genus
Source: Genome Biol. 2008 Mar 3;9(3):R49. doi: 10.1186/gb-2008-9-3-r49 (PMC2397501; doi:10.1186/gb-2008-9-3-r49)
Supplement: Additional data file 10 — The number of genes that failed the ILD test with the target gene at P < 0.01 for total, intron, exon and the third codon sites, respectively, and the P value of the ILD test between the target gene and all the rest of genes. [file gb-2008-9-3-r49-S10.pdf]

Additional data file 10. Number of genes that failed the ILD test with the target gene at *P*<0.01 for total, intron, exon and the 3rd codon sites, respectively, and the *P* value of ILD test between the target gene and all the rest of genes. Each of 39 genes (bold face) were significantly incongruent with the remaining genes (*P*<0.01).

ID [*]	Total	Intron	Exon	3rd codon	*P* value
**9-05**	121	102	46	13	0.001
**4-01**	119	72	89	66	0.001
**8-08**	119	114	53	12	0.001
**11-07**	105	84	21	14	0.001
**2-03**	104	80	0	0	0.001
**11-05**	103	87	0	0	0.001
**10-08**	97	84	0	0	0.001
**6-05**	93	78	5	2	0.001
**11-08**	93	81	4	2	0.001
**3-08**	92	84	2	1	0.007
**7-01**	92	76	16	10	0.001
**11-12**	89	76	1	0	0.001
8-04	84	69	9	6	0.454
9-09	83	56	22	13	0.096
**5-03**	82	70	1	0	0.001
**11-02**	78	88	11	13	0.001
**6-10**	77	81	4	4	0.002
**9-04**	75	63	1	0	0.001
**7-08**	74	60	0	0	0.001
**7-07**	73	46	1	1	0.001
**6-07**	68	42	24	18	0.001
**4-05**	67	61	8	0	0.001
3-13	66	57	16	9	0.999
**12-06**	65	25	18	2	0.002
**6-12**	63	54	0	0	0.001
1-05	59	43	1	0	0.214
4-09	56	21	25	16	0.26
**9-02**	55	5	9	7	0.03
**5-11**	52	--- [†]	17	14	0.001
**1-10**	50	36	0	0	0.001
2-08	50	46	11	5	1
**5-07**	50	35	2	1	0.01
**11-11**	50	34	2	0	0.004
**12-02**	50	46	12	2	0.001
1-01	47	38	0	0	0.399
**5-12**	47	26	9	2	0.001

**7-02**	47	36	1	1	0.044
4-10	45	46	1	1	0.079
8-12	44	43	4	1	0.064
1-02	43	30	1	1	0.999
11-06	43	35	3	1	1
**6-13**	41	31	13	10	0.046
1-14	40	35	3	2	0.196
3-01	39	24	6	1	0.094
4-08	39	36	11	5	0.447
9-06	39	32	1	0	0.637
**10-01**	38	26	0	0	0.005
3-06	37	24	6	1	0.066
**8-06**	37	26	1	0	0.001
**12-01**	37	18	3	1	0.007
3-07	36	10	4	5	0.077
9-08	36	29	8	5	0.999
7-06	35	13	6	6	0.138
8-05	35	26	0	4	0.393
**10-02**	35	30	0	0	0.001
12-08	35	24	2	1	0.772
6-08	34	22	1	0	0.316
8-11	34	19	2	1	0.415
8-10	33	17	3	1	0.578
4-06	32	20	1	0	1
11-10	32	19	11	0	0.41
2-01	31	31	3	1	0.409
2-02	31	19	0	0	0.592
2-07	31	9	0	0	0.261
9-10	30	5	2	1	0.178
4-07	29	29	2	1	1
6-02	29	26	1	0	0.205
12-03	29	30	0	0	0.242
12-04	29	21	2	1	1
**1-04**	28	14	0	0	0.049
1-16	28	32	4	2	0.717
5-09	28	25	5	3	1
10-05	28	32	2	1	1
3-04	27	23	6	3	0.224
5-10	27	8	5	1	1
6-03	26	6	3	2	0.598
12-05	26	8	2	2	0.266
6-11	25	13	6	1	1
11-09	25	26	0	0	1
1-06	24	32	4	1	1

2-04	24	36	4	5	1
4-02	24	6	3	3	1
10-07	24	20	0	0	1
**1-12**	23	24	3	3	0.032
4-03	22	25	4	3	0.758
2-05	21	---	5	1	0.439
5-13	20	12	1	1	0.18
10-10	20	7	0	0	1
3-09	19	25	1	1	0.626
3-05	18	12	3	2	0.797
4-04	18	9	5	1	0.625
5-04	18	5	0	0	0.811
6-06	18	7	31	5	0.056
9-07	18	18	3	2	0.236
1-07	17	25	2	1	0.118
3-03	17	17	2	4	1
**3-11**	17	6	0	0	0.025
4-11	17	4	5	3	0.163
10-11	17	17	---	---	0.799
11-04	17	21	1	1	1
1-08	16	7	1	0	1
5-08	15	11	2	0	0.118
7-04	15	16	0	0	0.999
8-03	15	18	3	1	0.423
11-03	15	3	5	1	0.114
1-09	13	12	2	0	0.293
2-09	13	3	2	5	1
5-06	12	13	1	0	0.239
5-14	12	12	0	0	0.077
1-13	11	5	0	0	0.784
1-15	11	11	0	0	0.074
4-12	11	6	0	0	0.431
6-04	11	4	2	4	0.782
9-01	11	6	0	0	1
3-10	10	4	0	0	0.495
6-09	10	---	2	1	0.227
3-12	9	3	3	1	0.596
7-05	9	7	5	2	0.601
9-03	9	2	0	0	0.244
11-13	9	2	0	0	0.356
1-17	8	---	3	0	1
5-05	8	7	3	1	0.178
7-09	7	12	3	2	0.334
10-04	7	3	0	0	0.354

11-01	7	10	3	1	0.458
1-03	6	5	12	1	1
2-06	6	3	0	0	0.62
8-07	6	5	3	2	0.709
8-09	6	5	0	0	0.209
1-11	5	3	0	0	0.759
3-02	5	0	0	0	0.775
8-01	5	3	1	0	1
5-02	4	4	2	3	0.44
6-01	4	3	1	1	1
10-06	4	9	2	1	0.807
5-01	2	---	0	0	0.55
8-02	2	0	0	0	0.627
12-07	2	1	1	0	1
10-03	1	1	0	0	1
12-09	1	1	0	0	1
7-03	0	0	0	0	1
10-09	0	0	0	0	1

[*] Numbers indicates the relative location on rice chromosomes of the genes (Additional data file 1) and arranged by the degree of significant incongruence each has with the other 141 genes in descending order.

[†] not available
