# Supplementary material for: Analysis of 142 genes resolves the rapid diversification of the rice genus
Source: Genome Biol. 2008 Mar 3;9(3):R49. doi: 10.1186/gb-2008-9-3-r49 (PMC2397501; doi:10.1186/gb-2008-9-3-r49)
Supplement: Additional data file 11 — Topologies of bootstrap 75% majority-rule consensus trees by different methods of analyses for each gene. [file gb-2008-9-3-r49-S11.pdf]

Additional data file 11. The topologies of bootstrap 75% majority-rule consensus trees by different methods of analyses for each gene.

ID*	ML	MP	ML (RY-coding)	NJ (LogDet distance)
1-01	((((A,B),C),E),F,G),L)	(((((A,B),C),E),G),F),L)	(((A,B),C),E,F,G),L)	((((A,B),C),E),F,G),L)
1-02	((((A,B),C,E),F),G),L)	((((A,B),C,E),F),G),L)	((((A,B),C,E),F),G),L)	((((A,B),C,E),F),G),L)
1-03	(((A,B,C),E),F,G),L)	(((A,B,C),E),F,G),L)	((A,B,C,E),F,G),L)	((((A,B),C),E),F,G),L)
1-04	((A,B,C,E),(F,G)),L)	((A,B,C,E),(F,G)),L)	((A,(B,C,E)),F,G),L)	((A,B,C,E),F,G),L)
1-05	(((A,(B,C),E),F),G),L)	(((A,((B,C),E)),F),G),L)	((A,B,C,E,F),G),L)	(((A,B,C,E),F),G),L)
1-06	((((A,B),C),E),F,G),L)	((((A,B),C),E),F,G),L)	(((A,B),C,E),F,G),L)	((((A,B),C),E),F,G),L)
1-07	((A,B,C,E),F,G),L)	((A,(B,C,E)),F,G),L)	((A,B,C,E),G,F),L)	((A,B,C,E),F,G),L)
1-08	(((A,B),C),E),L)	(((A,B),C),E),L)	((A,B,C,E),L)	((A,B,C,E),L)
1-09	(((A,B,C),E),F,G),L)	((A,B,C,E),F,G),L)	(((A,B,C),E),F,G),L)	(((A,B,C),E),F,G),L)
1-10	(((A,C),(B,E)),G),L)	(((A,C),B,E),G),L)	(((A,C),B,E),G),L)	(((A,C),B,E),G),L)
1-11	((A,B),C,E),F)	((A,B,C,E),F)	((A,B),C,E),F)	((A,B,C,E),F)
1-12	((((A,B,C),E),G),F),L)	((((A,B,C),E),G),F),L)	(((A,B,C,E),G),F),L)	((((A,B,C),E),G),F),L)
1-13	((A,B),C,E),L)	((A,B),C,E),L)	((A,B,E,C),L)	((A,B),C,E),L)
1-14	((((A,C),B),E),F,G),L)	((((A,C),B),E),F,G),L)	((A,C,B),E,F,G),L)	((((A,C),B),E),F,G),L)
1-15	((A,B,C,E),F,G),L)	(((A,B,C,E),G),F),L)	((A,B,C,E),F,G),L)	((A,B,C,E,G),F),L)
1-16	(((A,B),C,E),F,G),L)	(((A,B),C),E,F,G),L)	(((A,B),C,E),F,G),L)	(((A,B),C,E),F,G),L)
1-17	((A,B,C,E,F,G),L)	((A,B),C,E,F,G),L)	((A,C,E,F,G,B),L)	(((A,B),C),E,F,G),L)
2-01	(((A,B),(C,E)),F,G),L)	(((A,B),(C,E)),F,G),L)	((A,B,(C,E)),F,G),L)	(((A,B),(C,E)),F,G),L)
2-02	((((A,B,C),E),F),G),L)	((((A,B,C),E),F),G),L)	((((A,B,C),E),F),G),L)	(((A,B,C),E),F,G),L)
2-03	((((A,E),(B,C)),F),G),L)	((((A,E),(B,C)),F),G),L)	((A,E),(B,C),F,G),L)	(((A,E),(B,C)),F,G),L)
2-04	(((A,B,C),E),F,G),L)	(((A,B,C),E),F,G),L)	(((A,B,C),E),F,G),L)	(((A,B,C),E),F,G),L)
2-05	(((A,B,C),E),(F,G)),L)	((A,B,C,E),(F,G)),L)	((A,B,C,E,F,G),L)	(((A,B,C),E),(F,G)),L)
2-06	(((A,C),B,E),F),L)	((A,B,C,E),F),L)	(((A,C),B,E),F),L)	((A,B,C,E),F),L)
2-07	((A,B,C,E,F,G),L)	((A,B,C,E),F,G),L)	((A,B,C,E,F,G),L)	((A,B,C,E),F,G),L)

2-08	(((((A,B,C),E),F),G),L)	((((A,B,C,E),F),G),L)	((((A,B,C,E),F),G),L)	((((A,B,C,E),F),G),L)
2-09	((((A,B,C),E),F,G),L)	((((A,B,C),E),F,G),L)	((((A,B,C),E),F,G),L)	(((((A,B,C),E),F),G),L)
3-01	((((A,(B,C),E),F),G),L)	(((A,(B,C),E),F,G),L)	(((A,(B,C),E),F,G),L)	((((A,(B,C),E),F),G),L)
3-02	(((A,B,C,E),F),L)	(((A,B,C,E),F),L)	(((A,B,C,E),F),L)	(((A,B,C,E),F),L)
3-03	((((A,B),C),E,F,G),L)	((((A,B),C),E,F,G),L)	((A,B,C,E,F,G),L)	((((A,B),C),E,F,G),L)
3-04	(((((A,C),B),E),F,G),L)	(((((A,C),B),E),F,G),L)	((((A,C),B,E),F,G),L)	((((A,C),B,E),(F,G)),L)
3-05	((((A,B,C,E),F),G),L)	((((A,B,C,E),F),G),L)	((((A,B,C,E),F),G),L)	((((A,B,C,E),F),G),L)
3-06 †	(((((A,B,C),E),G),F),L)	(((((A,B,C),E),G),F),L)	((((A,B,C,E),F),G),L)	(((((A,B,C),E),G),F),L)
3-07	((((A,B,C),E),F,G),L)	(((((A,B,C),E),G),F),L)	(((A,B,C),E,F,G),L)	((((A,B,C),E),F,G),L)
3-08	(((((A,C),(B,E)),F),G),L)	(((((A,C),(B,E)),F),G),L)	(((A,C,(B,E)),F,G),L)	(((A,(B,E),C),F,G),L)
3-09	(((((A,C),B),E),F,G),L)	(((((A,C),B),E),F,G),L)	((((A,C,B),E),F,G),L)	(((((A,C),B),E),F,G),L)
3-10	((A,(B,C)),E)	((A,(B,C)),E)	((A,B,C),E)	((A,(B,C)),E)
3-11	(((A,C),B),E)	(((A,C),B),E)	(((A,C),B),E)	(((A,C),B),E)
3-12	(((A,B,C,E),F,G),L)	(((A,B,C,E),F,G),L)	(((A,B,C,E),F,G),L)	(((A,B,C,E),F,G),L)
3-13	(((((A,B,C),E),F),G),L)	(((((A,B,C),E),F),G),L)	(((((A,B,C),E),F),G),L)	(((((A,B,C),E),F),G),L)
4-01	((((A,G),B,C,E),F),L)	((((A,G),B,C,E),F),L)	((((A,G),B,C,E),F),L)	((((A,G),B,C,E),F),L)
4-02	(((((A,B),C),E),F,G),L)	(((((A,B),C),E),F,G),L)	(((A,B,C),E,G,F),L)	(((((A,B),C),E),F,G),L)
4-03	((((A,B,C),E),F,G),L)	((((A,B,C),E),F,G),L)	((((A,B,C,E),F),G),L)	((((A,B,C),E),F,G),L)
4-04	(((((A,B),C),E),(F,G)),L)	(((((A,B),C),E),F,G),L)	(((A,B,C,E),(F,G)),L)	(((((A,B),C),E),(F,G)),L)
4-05	((((A,(B,E)),C),F,G),L)	((((A,B,E),C),F,G),L)	((((A,(B,E)),C),F,G),L)	((((A,B,E),C),F,G),L)
4-06	((((((A,B),C),E),F),G),L)	((((((A,B),C),E),F),G),L)	(((((A,B,C),E),F),G),L)	(((((A,B,C),E),F),G),L)
4-07	(((((A,B),C),E),F),G)	(((((A,B),C),E),F),G)	(((((A,B),C),E),F),G)	(((((A,B),C),E),F),G)
4-08	(((((A,C),B),E,F),G),L)	((((A,C),B),E,F,G),L)	(((A,B,C),E,F,G),L)	(((((A,C),B),E,F),G),L)
4-09	(((((A,B),C,E),F),G),L)	((((((A,B),E),C),F),G),L)	((((A,B),C,E),F,G),L)	(((((A,B),C,E),F),G),L)
4-10	(((((A,(B,C)),E),F),G),L)	((((A,(B,C),E),F),G),L)	((A,(B,C),E,F,G),L)	((((A,(B,C),E),F),G),L)
4-11	((((A,B,C,E),G),F),L)	((((A,B,C,E),G),F),L)	(((A,B,C,E),F,G),L)	(((A,B,C,E),F,G),L)
4-12	((((A,B),C,E),F,G),L)	(((A,B,C,E),F,G),L)	((((A,B),(C,E)),F,G),L)	(((A,B,C,E),F,G),L)
5-01	(((A,B,C,E,G),F),L)	((A,B,C,E,F,G),L)	((A,B,C,E,G,F),L)	((((A,B,C,E),G),F),L)

5-02	(((A,B,C,E),F,G),L)	(((A,B,C,E),F,G),L)	((A,B,E,C,F,G),L)	(((A,B,C,E),F,G),L)
5-03	(((((A,B),E),C),F,G),L)	((((((A,B),E),C),G),F),L)	((A,B,E,C,F,G),L)	((((A,B,E),C),F,G),L)
5-04	((((A,B),C,E),F),G)	(((A,B,(C,E)),F),G)	(((A,B,C,E),F),G)	(((A,B,(C,E)),F),G)
5-05	(((A,B,C,E),F,G),L)	(((A,B,C,E),F,G),L)	(((A,B,C,E),F,G),L)	(((A,B,C,E),F,G),L)
5-06	(((A,B,(C,E)),F,G),L)	(((A,B,(C,E)),F,G),L)	(((A,B,(C,E)),G,F),L)	(((A,(B,(C,E))),F,G),L)
5-07	(((A,B,C,E),(F,G)),L)	(((A,B,C,E),(F,G)),L)	(((A,E,C,B),(F,G)),L)	(((A,B,C,E),(F,G)),L)
5-08	((((A,B,C,E),G),F),L)	((((A,B,C,E),G),F),L)	((((A,B,C,E),G),F),L)	(((A,B,C,E),F,G),L)
5-09	(((((A,B),C),E),F,G),L)	(((((A,B,C),E),F),G),L)	((((A,B),C,E),F,G),L)	((((A,B,C),E),F,G),L)
5-10	(((((A,B),C),E),F,G),L)	((((((A,B),C),E),F),G),L)	(((A,B,C,E),F,G),L)	(((((A,B),C),E),F,G),L)
5-11	(((A,B,C,E,G),F),L)	(((A,B,C,E,G),F),L)	(((A,B,C,E,G),F),L)	(((A,B,C,E,G),F),L)
5-12 †	(((A,C),B,E),F)	(((A,C),B,E),F)	((((A,C),E),B),F)	(((A,C),(B,E)),F)
5-13	(((A,C,E),(F,G)),L)	((((A,E),C),(F,G)),L)	(((A,C,E),(F,G)),L)	(((A,C,E),(F,G)),L)
5-14	(((A,C),B),E)	(((A,C),B),E)	(((A,C),B),E)	(((A,C),B),E)
6-01	(((A,B,C),F,G),L)	(((A,B,C),F,G),L)	(((A,B,C),(F,G)),L)	((((A,B),C),F,G),L)
6-02	((((A,B,C,E),F),G),L)	((((A,B,C,E),F),G),L)	(((A,B,C,E),F,G),L)	((((A,B,C,E),F),G),L)
6-03	((((A,B),C,E),(F,G)),L)	((((A,B),C,E),F,G),L)	(((A,B,C,E),(F,G)),L)	(((A,B,C,E),(F,G)),L)
6-04	(((((A,B),C),E),F,G),L)	(((((A,B),C),E),F,G),L)	((((A,B,C),E),F,G),L)	((((A,B,C),E),F,G),L)
6-05	(((A,(B,C),E),(F,G)),L)	(((A,(B,C),E),(F,G)),L)	(((A,(B,C),E),(F,G)),L)	(((A,(B,C),E),(F,G)),L)
6-06	(((A,B,C,E),(F,G)),L)	(((A,B,C,E),(F,G)),L)	((A,B,C,E,F,G),L)	(((A,B,C,E),(F,G)),L)
6-07	((((A,E),(B,C)),F,G),L)	(((A,(B,C),E),F,G),L)	(((A,E,(B,C)),F,G),L)	(((((A,E),(B,C)),G),F),L)
6-08	((((A,B,C),E),F,G),L)	(((((A,B,C),E),G),F),L)	(((A,B,C),E,F,G),L)	((((((A,C),B),E),G),F),L)
6-09	(((A,B,C,E),F,G),L)	(((A,B,C,E),F,G),L)	(((A,C,B,E,F),G),L)	((((A,C),B,E),F,G),L)
6-10	(((((A,C),(B,E)),F),G),L)	(((((A,C),(B,E)),F),G),L)	((((A,C),(B,E)),F,G),L)	((((A,C),B,E),F,G),L)
6-11 †	((((A,B,C),E),F,G),L)	(((((A,B,C),E),F),G),L)	(((A,C,B,E),(F,G)),L)	((((((A,C),B),E),F),G),L)
6-12	((A,(B,C,E)),G)	((A,(B,C,E)),G)	((A,(B,C,E)),G)	((A,(B,(C,E))),G)
6-13	(((((A,B,E),C),G),F),L)	(((((A,B,E),C),G),F),L)	((((A,B,E,C),G),F),L)	(((((A,B,E),C),G),F),L)
7-01	(((A,(B,(C,E))),F),G)	(((A,(B,(C,E))),F),G)	((A,(B,(C,E)),F),G)	((A,(B,(C,E)),F),G)
7-02	(((((A,B,C),E),G),F),L)	((((((A,B),C),E),G),F),L)	((((((A,B),C),E),G),F),L)	((((((A,B),C),E),G),F),L)

7-03	((A,B,C),F)	((A,B,C),F)	((A,C,B),F)	((A,B,C),F)
7-04	(((A,B,C),E),F)	(((A,B,C),E),F)	(((A,B,C),E),F)	(((A,B,C),E),F)
7-05	(((A,B,C,E),F,G),L)	(((A,B,C,E),F,G),L)	(((A,B,C,E),F,G),L)	((((A,B,C,E),F),G),L)
7-06	(((((A,C),B,E),F),G),L)	(((((A,C),B,E),F),G),L)	((((A,C,B,E),F),G),L)	(((((A,C),B,E),F),G),L)
7-07	((((A, (B, (C,E))),G),F),L)	((((A, (B, (C,E))),G),F),L)	(((A, (B,C,E)),G,F),L)	((((A,B,C,E),G),F),L)
7-08	((((A,C), (B,E)),F,G),L)	((((A,C), (B,E)),F,G),L)	(((((A,C), (B,E)),G),F),L)	((((A,C), (B,E)),F,G),L)
7-09	(((A,B,C,E),F,G),L)	(((A,B,C,E),F,G),L)	(((A,B,C,E),F,G),L)	(((A,B,C,E),F,G),L)
8-01	((((A,B,C,E),F),G),L)	(((A,B,C,E),F,G),L)	(((A,B,C,E,F),G),L)	(((A,B,C,E),F,G),L)
8-02	(((A,B,C),F),L)	(((A,B,C),F),L)	(((A,B,C),F),L)	(((A,B,C),F),L)
8-03	(((A,B,C,E),F,G),L)	(((A,B,C,E),F,G),L)	(((A,B,C,E),F,G),L)	((((A,B,C),E), (F,G)),L)
8-04	(((((A, (B,C)),E),F),G),L)	(((((A, (B,C)),E),F),G),L)	(((((A,B,C),E),F),G),L)	(((((A,B,C),E),F),G),L)
8-05	(((A,B,C),E,F,G),L)	(((A, (B,C)),E,F,G),L)	((A,B,C,E,F,G),L)	(((A,B,C), (E,F),G),L)
8-06	(((A,C),B),G)	(((A,C),B),G)	(((A,C),B),G)	(((A,C),B),G)
8-07	((((A,B,C),E),F),G)	(((A,B,C,E),F),G)	((((A,B,C),E),F),G)	(((A,B,C,E),F),G)
8-08	(((A,G),B,C,E,F),L)	(((((A,G),C),B,E),F),L)	(((A,G),C,B,E,F),L)	(((((A,G),C),B,E),F),L)
8-09	((((A,C),B),F,G),L)	((((A,C),B),F,G),L)	(((A,C,B),F,G),L)	((((A,C),B),F,G),L)
8-10	(((((A,B),C),E), (F,G)),L)	(((((A,B),C),E),F,G),L)	((A,B,C,E, (F,G)),L)	((((A,B,C),E),F,G),L)
8-11	(((((A,B),C),E),F,G),L)	(((((A,B),C),E),F,G),L)	((((((A,B),C),E),G),F),L)	(((((A,B),C),E),F,G),L)
8-12	(((((A,B,C),E),G),F),L)	(((((A,B,C),E),G),F),L)	(((((A,B,C),E),G),F),L)	(((((A,B,C),E),G),F),L)
9-01	((((A,B,C),E),F,G),L)	(((A,B,C,E),F,G),L)	(((((A,B,C),E),F),G),L)	(((A,B,C,E),F,G),L)
9-02	(((A,B,C,E),F,G),L)	((((A, (B, (C,E))),G),F),L)	((A,B,C,F,G,E),L)	(((A,B,C,E),F,G),L)
9-03	(((A,B,C), (F,G)),L)	(((A,B,C),F,G),L)	(((A,B,C), (F,G)),L)	(((A, (B,C)), (F,G)),L)
9-04	(((A,B,C,E), (F,G)),L)	(((A,B,C,E), (F,G)),L)	((A,B,C,E, (F,G)),L)	(((A,B,C,E), (F,G)),L)
9-05	((((A,B),E),C, (F,G)),L)	((((A,B),E),C, (F,G)),L)	((((A,B),E),C, (F,G)),L)	((((A,B),E),C, (F,G)),L)
9-06	((((A,B,C),G),E),L)	(((A,B,C),E,G),L)	((((A,B,C),G),E),L)	(((((A,B),C),G),E),L)
9-07	((((A,B),C,E),F,G),L)	(((A,B,C,E),F,G),L)	(((A,B,C,E),F,G),L)	(((A,B,C,E),F,G),L)
9-08	(((((A,B),C),E),F,G),L)	(((((A,B),C),E),F,G),L)	((((A,B,C),E),F,G),L)	(((((A,B),C),E),F,G),L)
9-09	((((((A,C),B),E),F),G),L)	((((((A,C),B),E),F),G),L)	((((((A,C),B),E),F),G),L)	((((((A,C),B),E),F),G),L)

9-10	(((((A, C, E), B), F), G), L)	((((A, C, E), B), F, G), L)	((((A, C, E), B), G, F), L)	(((((A, C, E), B), F), G), L)
10-01	((((A, B, C, E), G), F), L)	((((A, B, C, E), G), F), L)	(((A, B, C, E), F, G), L)	((((A, B, C, E), G), F), L)
10-02	(((A, C), B), E)	(((A, C), B), E)	(((A, C), B), E)	(((A, C), B), E)
10-03	((A, B, C), E)	((A, B, C), E)	((A, B, C), E)	((A, B, C), E)
10-04	((((A, C, E), B), G), L)	((((A, C, E), B), G), L)	((((A, C, E), B), G), L)	((((A, C, E), B), G), L)
10-05	(((((A, B), C), E), F), G)	(((((A, B), C), E), F), G)	((((A, B, C), E), F), G)	(((((A, B), C), E), F), G)
10-06	((((A, B, C, E), F), G), L)	(((A, B, C, E), F, G), L)	((((A, B, C, E), F), G), L)	((((A, (B, C)), E), F, G), L)
10-07	(((((A, B, C), E), F), G), L)	((((A, B, C), E), F, G), L)	(((A, B, C), E, F, G), L)	((((A, B, C), E), F, G), L)
10-08	(((((A, E), C), B, G), F), L)	(((((A, E), C), B, G), F), L)	(((A, E, C), B, F, G), L)	(((((A, E), B, C), G), F), L)
10-09	((A, B, C, E), L)	((A, B, C, E), L)	((A, B, C, E), L)	((A, B, C, E), L)
10-10	(((A, B), C), E)	(((A, B), C), E)	(((A, B), C), E)	(((A, B), C), E)
10-11 †	((((A, B), C, E), F, G), L)	((((A, B), C, E), F, G), L)	((((A, B), C, E), (F, G)), L)	((((A, B, C, E), F), G), L)
11-01	(((A, B, C, E), G), L)	(((A, B, C, E), G), L)	(((A, B, C, E), G), L)	(((A, B, C, E), G), L)
11-02	((((A, E), (B, C)), F, G), L)	(((A, E), (B, C), F, G), L)	(((A, E), (B, C), F, G), L)	(((A, E), (B, C), F, G), L)
11-03	((((A, B, C), E), F, G), L)	((((A, B, C), E), F, G), L)	((((A, B, C), E), F, G), L)	((((A, B, C), E), F, G), L)
11-04	((((A, B), C, E), F, G), L)	((((A, B), C, E), F, G), L)	(((A, B, C, E), F, G), L)	(((A, B, C, E), F, G), L)
11-05	((((A, C), (B, E)), (F, G)), L)	((((A, C), (B, E)), (F, G)), L)	((((A, C), (B, E)), F, G), L)	((((A, C), (B, E)), (F, G)), L)
11-06	(((((A, B), C), E), F), G)	(((((A, B), C), E), F), G)	(((((A, B), C), E), F), G)	(((((A, B), C), E), F), G)
11-07	(((((A, B), C, E), G), F), L)	(((((A, B), C, E), G), F), L)	(((((A, B), (C, E)), G), F), L)	(((((A, B), (C, E)), G), F), L)
11-08	(((A, E), (B, C), F, G), L)	(((A, E), (B, C), F, G), L)	(((A, E), (B, C), F, G), L)	(((A, E), (B, C), F, G), L)
11-09	(((A, B), C), E)	(((A, B), C), E)	(((A, B), C), E)	(((A, B), C), E)
11-10	((((A, B, C), E), F, G), L)	(((((A, B, C), E), G), F), L)	((((A, C, B), E), G, F), L)	(((((A, B, C), E), G), F), L)
11-11	((((A, (B, C)), E), (F, G)), L)	((((A, (B, C)), E), (F, G)), L)	(((A, (B, C), E), F, G), L)	(((A, (B, C), E), (F, G)), L)
11-12	((((A, B, E), C), (F, G)), L)	((((A, B, E), C), (F, G)), L)	(((A, B, C, E), (F, G)), L)	(((((A, B), E), C), (F, G)), L)
11-13	(((A, (B, C), E), F, G), L)	(((A, (B, C), E), F, G), L)	(((A, B, C, E), G, F), L)	(((A, (B, C), E), F, G), L)
12-01	((((A, B, E), C), F, G), L)	(((((A, B), E), C), F, G), L)	(((A, E, B, C), G, F), L)	(((((A, B), E), C), F, G), L)
12-02	((((A, (B, C)), E), (F, G)), L)	(((A, (B, C), E), (F, G)), L)	((((A, (B, C)), E), (F, G)), L)	((((A, (B, C)), E), (F, G)), L)
12-03	(((((A, B), C), G), F), L)	((((A, B), C), F, G), L)	(((((A, B), C), G), F), L)	((((A, B), C), F, G), L)

12-04	((((((A,B,C),E),F),G),L)	((((A,B,C),E),F),G),L)	((((A,B,C),E),F,G),L)	((((((A,B),C),E),F),G),L)
12-05	((((A, (B,C)),E),F,G),L)	((((A, (B,C)),E),F,G),L)	((((A, (B,C),E),G),F),L)	((((A, (B,C)),E),F,G),L)
12-06	(((A, (B,C,E)), (F,G)),L)	(((A,B,C,E), (F,G)),L)	((A, (B,C,E), (F,G)),L)	(((A,B,C,E), (F,G)),L)
12-07	(((A,B,C,E),G),L)	(((A,B,C,E),G),L)	(((A,B,C,E),G),L)	(((A,B,C,E),G),L)
12-08	((((((A,B,C),E),F),G),L)	((((A,B,C),E),F),G),L)	((((A,B,C),E),F,G),L)	((((A,B,C),E,F),G),L)
12-09	((A,B,C),E)	((A,B,C),E)	((A,B,C),E)	((A,B,C),E)

* Numbers are corresponding to gene's ID defined by authors in Additional data files 1 and 2.

† four out of 142 genes yielded incompatible topologies between different methods of analyses in terms of bootstrap 75% majority-rule consensus trees
