# Supplementary material for: Analysis of 142 genes resolves the rapid diversification of the rice genus
Source: Genome Biol. 2008 Mar 3;9(3):R49. doi: 10.1186/gb-2008-9-3-r49 (PMC2397501; doi:10.1186/gb-2008-9-3-r49)
Supplement: Additional data file 12 — Saturation analyses in the concatenated datasets of total, intron, exon, and third codon positions, respectively. [file gb-2008-9-3-r49-S12.pdf]

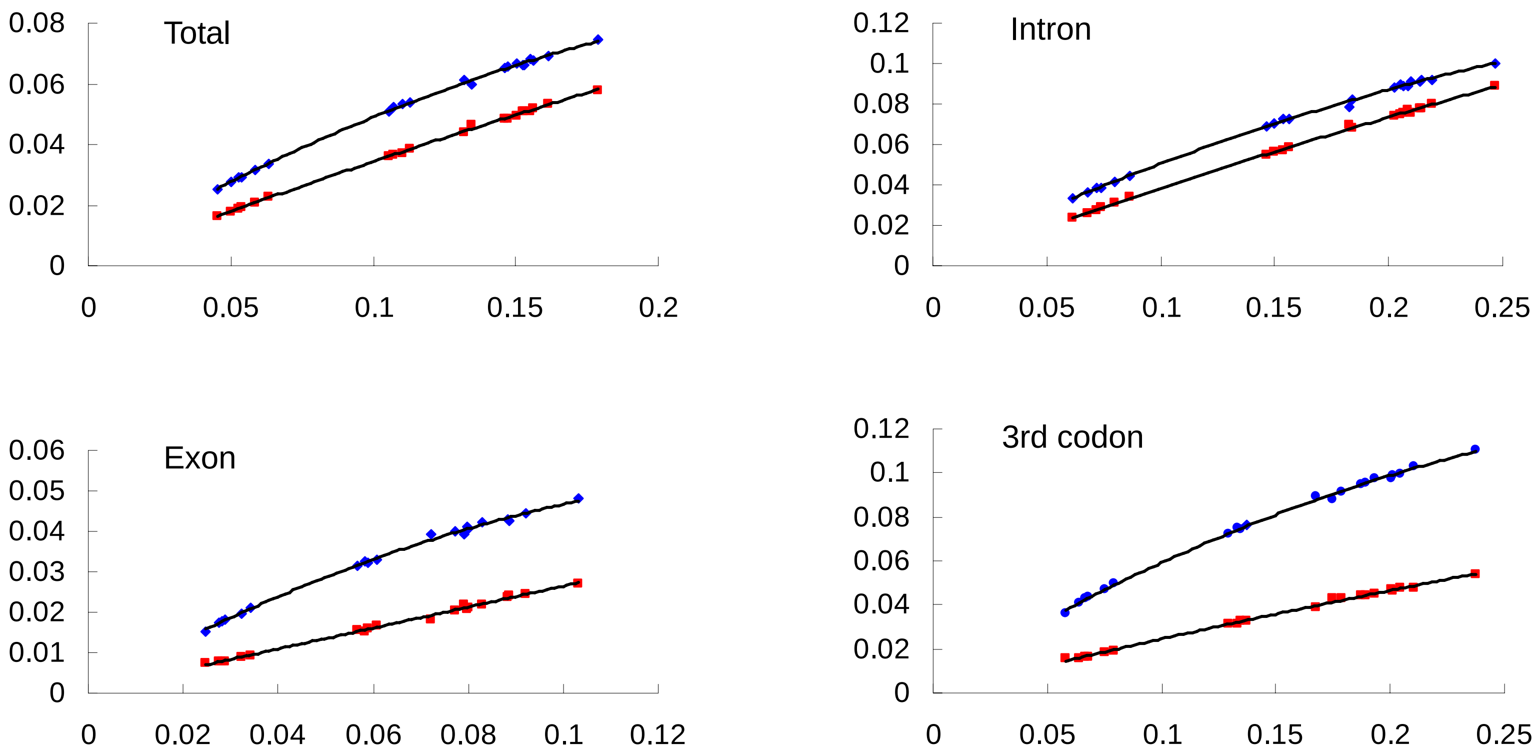

Additional data file 12. Saturation analyses in the concatenated datasets of total, intron, exon, and third codon positions, respectively. The x-axis represents corrected pairwise divergence estimated by maximum likelihood and the y-axis is the observed divergence (uncorrected *P*-distance). Transitions are shown as the blue diamonds and transversions are the red squares. A best-fit second degree polynomial is applied to describe the trend.
