# Supplementary material for: Analysis of 142 genes resolves the rapid diversification of the rice genus
Source: Genome Biol. 2008 Mar 3;9(3):R49. doi: 10.1186/gb-2008-9-3-r49 (PMC2397501; doi:10.1186/gb-2008-9-3-r49)
Supplement: Additional data file 13 — Proportions of topology (or clades) identical to those shown in Figure 1 inferred from randomly sampled genes or sites in 500 replicates. [file gb-2008-9-3-r49-S13.pdf]

Additional data file 13. The proportions of topology (or clades) identical to those shown in figure 1 inferred from randomly sampled genes or sites in 500 replicates. The capital letters A, B, C, F, and G represent *Oryza* genome types and R represents the rest of genome types including A, B, C, and E.

Size of random sample	ML method			MP method		
	Gene tree*	(AB)C	(RF)G	Gene tree*	(AB)C	(RF)G
20 genes	0.32	0.51	0.63	0.26	0.52	0.56
40 genes	0.45	0.58	0.77	0.35	0.61	0.60
60 genes	0.52	0.62	0.86	0.43	0.66	0.66
80 genes	0.66	0.72	0.92	0.57	0.78	0.73
100 genes	0.83	0.85	0.98	0.73	0.91	0.82
120 genes	0.97	0.97	1.00	0.87	0.98	0.90
10 kb	0.53	0.68	0.76	0.38	0.68	0.60
20 kb	0.67	0.79	0.86	0.56	0.83	0.70
30 kb	0.83	0.91	0.92	0.64	0.92	0.72
40 kb	0.91	0.95	0.96	0.75	0.97	0.80
50 kb	0.95	0.95	0.99	0.82	0.98	0.85
60 kb	0.99	0.99	1.00	0.88	1.00	0.90
70 kb	1.00	1.00	1.00	0.91	1.00	0.92
80 kb	1.00	1.00	1.00	0.96	1.00	0.96
90 kb	1.00	1.00	1.00	0.97	1.00	0.97
100 kb	1.00	1.00	1.00	0.98	1.00	0.99

* denotes the gene tree that has identical topology to figure 1.
