# Supplementary material for: Analysis of 142 genes resolves the rapid diversification of the rice genus
Source: Genome Biol. 2008 Mar 3;9(3):R49. doi: 10.1186/gb-2008-9-3-r49 (PMC2397501; doi:10.1186/gb-2008-9-3-r49)
Supplement: Additional data file 14 — Primers for PCR amplification and the GenBank accession numbers of the sequences of 142 loci sampled. [file gb-2008-9-3-r49-S14.pdf]

Additional data file 14. A list of the primers for PCR amplification and GenBank accession numbers of the sequences of 142 loci sampled in the present study.

ID *	Primer F	Primer R	GenBank accession number									
			O. rufipogon	*O. punctata*	*O. officinalis*	*O. australiensis*	*O. brachyantha*	*O. granulata*	*Leersia*	*O. barthii*	*O. rhizomatis*	*O. eichingeri*
1_01	TCAGCCCACCTTATGTGTTC	TGTGCAGCAACAACATCCAT	EF577622	EF577623	EF577624	EF577625	EF577626	EF577627	EF577628	---	---	---
1_02	CAAGCGCAAATGTGTTACTG	CCTTCCTTCCATCTTCAATG	EF578231	EF578232	EF578233	EF578234	EF578235	EF578236	EF578237	EU503438	EU503439	EU503440
1_03	CTGCGCCAACAAATCAGTAG	ATGGGCGTAATGCTGAGGCT	EF578245	EF578246	EF578247	EF578248	EF578249	EF578250	EF578251	EU503441	EU503442	EU503443
1_04	CACAAGGGCCATGTTTCAGC	CACTGCCAGAAACCAAATAG	EF578259	EF578260	EF578261	EF578262	EF578263	EF578264	EF578265	EU503444	EU503445	EU503446
1_05	TTCCGCTCTCGCTCCTCAAG	CATCAGGAACCCGAAGATAC	EF578333	EF578334	EF578335	EF578336	EF578337	EF578338	EF578339	EU503492	EU503493	EU503494
1_06	TCACAACGAGCTGGCAGTGG	TCGCTCCATAGTGGGCTCAG	EF577764	EF577765	EF577766	EF577767	EF577768	EF577769	EF577770	EU503495	EU503496	EU503497
1_07	TTGAAGAGGTCCCGTGTGGT	TTGCCTTGCGCTTCTTCTTC	EF577812	EF577813	EF577814	EF577815	EF577816	EF577817	EF577818	EU503498	EU503499	EU503500
1_08	TAAGGAGCCGTCTGGGTC	CGTCAGCCTCGGCTTCTG	EF578314	EF578315	EF578316	EF578317	--- †	---	EF578318	EU503447	EU503448	EU503449
1_09	CCGATGGCTAAAGATGGAGT	AGCTCCTGAATACGCCTAG	EF577559	EF577560	EF577561	EF577562	EF577563	EF577564	EF577565	---	---	---
1_10	TGTTAGCCCTCGTGGAGCAG	GTCCTCGGTCCTCGCATCTG	EF578075	EF578076	EF578077	EF578078	---	EF578079	EF578080	EU503501	EU503502	EU503503
1_11	TATGAGAAACTTGAGGAAGGG	CCCATGCATACACCACAACTG	EF577973	EF577974	EF577975	EF577976	EF577977	---	---	EU503504	EU503505	EU503506
1_12	TGATCTCAGTGGCAATGACC	TTCCCATCCCAGCTAGCATC	EF578167	EF578168	EF578169	EF578170	EF578171	EF578172	EF578173	---	---	---
1_13	CGAGGAGCATGTTGGATTTG	CCTTTCCTGGAGCTCGAATG	EF578191	EF578192	EF578193	EF578194	---	---	EF578195	---	---	---
1_14	AGCGTAGCTCTGAGCCATGA	GCCGATTTCAATAAGCTGAG	EF577525	EF577526	EF577527	EF577528	EF577529	EF577530	EF577531	---	---	---
1_15	GGAGGAATGAATGGTTACAT	CCACTGTCTTCATGCCATAG	EF578184	EF578185	EF578186	EF578187	EF578188	EF578189	EF578190	---	---	---
1_16	GGCGATAACATGGAACTTGG	ACCGAAAGGCTCAACAAGA	EF577580	EF577581	EF577582	EF577583	EF577584	EF577585	EF577586	---	---	---
1_17	ATGGCCACCTTCGAGCTSTA	TCRCAGGCCACRATCTTCAC	EF577601	EF577602	EF577603	EF577604	EF577605	EF577606	EF577607	---	---	---
2_01	GGAGCTTCGGTTTCTTCTGC	GGGGAATGTCCAGGGCACTG	EF577744	EF577745	EF577746	EF577747	EF577748	EF577749	EF577750	EU503531	EU503532	EU503533
2_02	GATGAAGTGCGCAAGATCTC	AATATAAAGACTTGGTTGCC	EF578203	EF578204	EF578205	EF578206	EF578207	EF578208	EF578209	---	---	---
2_03	TACACGCAGAGGCTGAAAGG	ATCGGGTCAGCAGGGAAGTC	EF577799	EF577800	EF577801	EF577802	EF577803	EF577804	EF577805	---	---	---

2_04	AACTTGTGGAGGTTAGCAG	AAGTCGCTCAGAGGCACG	EF577636	EF577637	EF577638	EF577639	EF577640	EF577641	EF577642	---	---	---
2_05	CAGGACGTTCATGTTCAGCAG	TCGTCGCCAAGCAGTTGTC	EF577664	EF577665	EF577666	EF577667	EF577668	EF577669	EF577670	---	---	---
2_06	GCTGGGCCTCAGACACCTAC	CTCTTAGTTTTTCACGCATATC	EF577806	EF577807	EF577808	EF577809	EF577810	---	EF577811	EU503348	EU503349	EU503350
2_07	GTTGGAGCTGAGGCTTGTGC	CCAGCGGTCGAGAACTTAAC	EF578252	EF578253	EF578254	EF578255	EF578256	EF578257	EF578258	EU503450	EU503451	EU503452
2_08	AGCTACGACTACGACCTGTT	CAGTTGCTATCAATATGTGC	EF577629	EF577630	EF577631	EF577632	EF577633	EF577634	EF577635	---	---	---
2_09	TTCTCGCCGTCGCAGTTCAC	GCAGTAGCACGCTTCCTCTC	EF578326	EF578327	EF578328	EF578329	EF578330	EF578331	EF578332	EU503453	EU503454	EU503455
3_01	ACCCGCTTCGCTGACCAATG	TGTCCCCAAAAGGCTCTGAG	EF577985	EF577986	EF577987	EF577988	EF577989	EF577990	EF577991	EU503507	EU503508	EU503509
3_02	ACGACAACTAGCAATTCTGG	ATTCATGCCGATAGTTGCTG	EF578042	EF578043	EF578044	EF578045	EF578046	---	EF578047	EU503510	EU503511	EU503512
3_03	TTGTTGGATTTGGGTGAGGC	GCAAAGCAAACAGGGCAAAC	EF578081	EF578082	EF578083	EF578084	EF578085	EF578086	EF578087	EU503513	EU503514	EU503515
3_04	TGGAATGAGCGAAGGAAGCC	GCGTGTTGATGCCGGGATTC	EF578146	EF578147	EF578148	EF578149	EF578150	EF578151	EF578152	EU503516	EU503517	EU503518
3_05	CCTGAAGCTGAAAACAAGAG	CTTTGTTGAGATCTTGCAGC	EF578347	EF578348	EF578349	EF578350	EF578351	EF578352	EF578353	EU503519	EU503520	EU503521
3_06	TCAAAAATCATCGGCGACAC	GAACTTCCACTTCCGCTCTC	EF577966	EF577967	EF577968	EF577969	EF577970	EF577971	EF577972	EU503432	EU503433	EU503434
3_07	AAGCCAAGGCACCGTTTC	GCTGCGGCCCAGGAAATC	EF577978	EF577979	EF577980	EF577981	EF577982	EF577983	EF577984	---	---	---
3_08	TGAAGGGAGCTGATGGACTG	AATAATCGAGCGCCCTTCAC	EF577992	EF577993	EF577994	EF577995	EF577996	EF577997	EF577998	EU503435	EU503436	EU503437
3_09	GTGGAAACAAAGGAGGTGTG	CCCTAAGATGCCCTCTGAAC	EF577999	EF578000	EF578001	EF578002	EF578003	EF578004	EF578005	---	---	---
3_10	CCTCCTGCGGATCTACGTTC	TGCTTTACCGGAGGCTTCAC	EF578006	EF578007	EF578008	EF578009	---	---	---	---	---	---
3_11	CTGTGATGCCCTGTTATGAT	GATAAGATCCTTCGCAGACT	EF578180	EF578181	EF578182	EF578183	---	---	---	---	---	---
3_12	GCCCTGATGGAACTCAAG	CAAGGCCTTTCAGATACTCA	EF578427	EF578428	EF578429	EF578430	EF578431	EF578432	EF578433	---	---	---
3_13	ATGAAGATACTGCAAAACGG	ATCTCATCTAGGCGAATGT	EF578406	EF578407	EF578408	EF578409	EF578410	EF578411	EF578412	---	---	---
4_01	ACGTCCATGCCCTATCCAAG	GTCCCTTGCGGCGTAATGAG	EF578340	EF578341	EF578342	EF578343	EF578344	EF578345	EF578346	---	---	---
4_02	CCACGAACACAGATTGGATTC	CCACATCTGATACGCATAAC	EF578273	EF578274	EF578275	EF578276	EF578277	EF578278	EF578279	EU503456	EU503457	EU503458
4_03	GAATTGTCAGATGAGGAGCC	ATACGACTAGGACCATCTGG	EF577573	EF577574	EF577575	EF577576	EF577577	EF577578	EF577579	---	---	---
4_04	TGGCAGAAGAAGTTACGAGG	CTTGGCAAATCTTTGACAAC	EF578266	EF578267	EF578268	EF578269	EF578270	EF578271	EF578272	EU503459	EU503460	EU503461
4_05	CCTCTGCTGATGGCTACTTG	GCCTGCAACCTTCTTCTAGT	EF577532	EF577533	EF577534	EF577535	EF577536	EF577537	EF577538	---	---	---
4_06	ACACTCAAGGTTCTCATGGC	TAGAGAGATCAGCCTTGTCC	EF578280	EF578281	EF578282	EF578283	EF578284	EF578285	EF578286	EU503462	EU503463	EU503464
4_07	CTGCTTTCTGGGGAGAGTC	AGTAGGAATAGGCACCAATG	EF578287	EF578288	EF578289	EF578290	EF578291	EF578292	---	EU503465	EU503466	EU503467

4_08	ACAGACACAAGATGCTTTGC	GAAGAGATGGTAGCACTTGG	EF578293	EF578294	EF578295	EF578296	EF578297	EF578298	EF578299	EU503468	EU503469	EU503470
4_09	TCCAGTTTCCGTGAATCAC	CCTTGACGACTGCATTCAC	EF577650	EF577651	EF577652	EF577653	EF577654	EF577655	EF577656	---	---	---
4_10	TGCAGCAACTTTTCTAAGAC	TAGGTCATTCTCGTCCAAAG	EF578307	EF578308	EF578309	EF578310	EF578311	EF578312	EF578313	EU503471	EU503472	EU503473
4_11	ATGTTGGATTGCCTGTGTTC	TGGGGATCAATAAGGCATTC	EF578300	EF578301	EF578302	EF578303	EF578304	EF578305	EF578306	EU503474	EU503475	EU503476
4_12	GGATTGTGGCAAGCAGGTC	ATGGCAGCGCGGTCGTAC	EF578319	EF578320	EF578321	EF578322	EF578323	EF578324	EF578325	EU503477	EU503478	EU503479
5_01	ATGGCCACCTTCGAGCTSTA	TCRCAGGCCACRATCTTCAC	EF577594	EF577595	EF577596	EF577597	EF577598	EF577599	EF577600	---	---	---
5_02	GCAAAGAGGCGCACTGCAGA	GATTTCATCATCCTTTGGTTC	EF578210	EF578211	EF578212	EF578213	EF578214	EF578215	EF578216	EU503480	EU503481	EU503482
5_03	AGGGTGACTTCCTGACCTTC	GCGCCATATTCTGCAAATCC	EF577771	EF577772	EF577773	EF577774	EF577775	EF577776	EF577777	EU503351	EU503352	EU503353
5_04	GAGCTTCAGTTCGCATAATCAG	TCGCGTACAACAATTTGCTTTC	EF577826	EF577827	EF577828	EF577829	EF577830	EF577831	---	EU503354	EU503355	EU503356
5_05	AGGGCCACCGAGGGTCCTAT	CACTGCCCAAGTCATTGAAG	EF577778	EF577779	EF577780	EF577781	EF577782	EF577783	EF577784	EU503357	EU503358	EU503359
5_06	GGCCAAAACTTGTGTTGAAATG	GAAGTGGATGGGCCTCTGTG	EF578196	EF578197	EF578198	EF578199	EF578200	EF578201	EF578202	EU503483	EU503484	EU503485
5_07	CAAAGATCCTATCCGCACAG	AAGCCCAGAGCGCCGATGAG	EF577792	EF577793	EF577794	EF577795	EF577796	EF577797	EF577798	EU503360	EU503361	EU503362
5_08	GGCGTTTCCCAATGTCCAAG	TATGAGAATCTCCTCCACTG	EF578217	EF578218	EF578219	EF578220	EF578221	EF578222	EF578223	EU503486	EU503487	EU503488
5_09	TGAAGAATCCTCCGCTGGAC	GCAGGTGGATCACGGTAACC	EF578224	EF578225	EF578226	EF578227	EF578228	EF578229	EF578230	EU503489	EU503490	EU503491
5_10	GCAAGAGTACGGACAAATGGTG	GCTTGCTGCTCTGGAAGTAG	EF577545	EF577546	EF577547	EF577548	EF577549	EF577550	EF577551	---	---	---
5_11	ATGCCCCCAGCCATTGGATG	GAAGCGACCCTTGGTGTC	EF578238	EF578239	EF578240	EF578241	EF578242	EF578243	EF578244	---	---	---
5_12	CACTATTGCCCTGCTACTGG	ATGAATCTGGAAGCCCAATG	EF578397	EF578398	EF578399	EF578400	EF578401	---	---	---	---	---
5_13	TGACAGTGATTTTGCTTACG	CAATGTCCGCTTCATCGAAC	EF578354	---	EF578355	EF578356	EF578357	EF578358	EF578359	---	---	---
5_14	AGGTCTCAGGATTACAACGC	GACCAGCTTTTCGCAATG	EF578402	EF578403	EF578404	EF578405	---	---	---	---	---	---
6_01	GCTGCAAGTTGCCCAGAAAT	CATGTGATTCACCACCTCTG	EF577758	EF577759	EF577760	---	EF577761	EF577762	EF577763	EU503522	EU503523	EU503524
6_02	CTGTCGCTGGATTTGGTTGG	CAAGGAACAGGTATCAACTC	EF577518	EF577519	EF577520	EF577521	EF577522	EF577523	EF577524	---	---	---
6_03	CATCCACAACATCTCCTACCAGG	CCAGTCTTGCCTTCGATGA	EF577608	EF577609	EF577610	EF577611	EF577612	EF577613	EF577614	---	---	---
6_04	CTGATTGTCAATTTTGGCATAG	CCCCAACATCATCTCCAGAAAG	EF577671	EF577672	EF577673	EF577674	EF577675	EF577676	EF577677	EU503525	EU503526	EU503527
6_05	GTTTGTTGTGAATGATTGGC	TCTGTGGATGGGTTCCAATC	EF577657	EF577658	EF577659	EF577660	EF577661	EF577662	EF577663	---	---	---
6_06	CCCTCGTCATGGCCTTCCT	TCGATGTTGCGGTCCAAGC	EF577730	EF577731	EF577732	EF577733	EF577734	EF577735	EF577736	EU503528	EU503529	EU503530
6_07	AAGCGTGCAGAACTTGGTTA	CCACAGGAGCAGCGAGGTTG	EF577737	EF577738	EF577739	EF577740	EF577741	EF577742	EF577743	---	---	---

6_08	AGCGACGCCTTCTACCACTG	GGGCAAGGACCATTCAAGTG	EF578017	EF578018	EF578019	EF578020	EF578021	EF578022	EF578023	---	---	---
6_09	CTGTAGTTGTGCTCAATTC	GCCATCGTCTTGTACATGTC	EF577643	EF577644	EF577645	EF577646	EF577647	EF577648	EF577649	---	---	---
6_10	GAACCTCCCACCAAATGTCG	TCAGAAATAGTCCCGCTCTT	EF577751	EF577752	EF577753	EF577754	EF577755	EF577756	EF577757	---	---	---
6_11	ACATGCCCAATTCAAATGAG	CAACCCTACTCGCCACATTC	EF578010	EF578011	EF578012	EF578013	EF578014	EF578015	EF578016	---	---	---
6_12	CCCAACATCTTAATTCACGA	AGCCAGAGAAAGCGCAAGAG	EF578367	EF578368	EF578369	EF578370	---	EF578371	---	---	---	---
6_13	GCGATAATTGTCCCTGGAAT	CTTCTGCCCAAGGGACGCAT	EF577566	EF577567	EF577568	EF577569	EF577570	EF577571	EF577572	---	---	---
7_01	GGGCTAATGTCACAAGATGG	CGGAATGCAATGGCCTTCTG	EF578174	EF578175	EF578176	EF578177	EF578178	EF578179	---	---	---	---
7_02	CCGGGTTCTATCTTGTTCTG	ACAGGACCACAACGGAAAAT	EF578160	EF578161	EF578162	EF578163	EF578164	EF578165	EF578166	---	---	---
7_03	ATATCTATAACCGGGATTGG	CCTTGCATATAAACTTTGCC	EF577715	EF577716	EF577717	---	EF577718	---	---	---	---	---
7_04	CAGTTGAGGCAGAGACTTGG	GCTTCTTGCACCCTATTGGA	EF577678	EF577679	EF577680	EF577681	EF577682	---	---	---	---	---
7_05	GAGAAGTGGTAGGGGGAAAG	TCCCGCTTGTGTAAACATTC	EF577853	EF577854	EF577855	EF577856	EF577857	EF577858	EF577859	EU503363	EU503364	EU503365
7_06	GGGAGATGGCGGTTTCTTTC	ACGCAACAATGTCGCAGCTC	EF577867	EF577868	EF577869	EF577870	EF577871	EF577872	EF577873	EU503366	EU503367	EU503368
7_07	TGGAGGAGGAGCTGTCATTC	CTGGTCCAGCTTCCTTTGTG	EF577819	EF577820	EF577821	EF577822	EF577823	EF577824	EF577825	EU503369	EU503370	EU503371
7_08	TTGCCCGTGTTTGGATTCAG	CAACCCGTGAGTCTCTTGTC	EF577839	EF577840	EF577841	EF577842	EF577843	EF577844	EF577845	EU503372	EU503373	EU503374
7_09	CCTGGATCTTTTTGGTGATGAG	TTCTTGATCCCGTAACCCACAG	EF577832	EF577833	EF577834	EF577835	EF577836	EF577837	EF577838	EU503375	EU503376	EU503377
8_01	TTCGGAAAGCTTCTAGTGTC	ACATCATGCCGTACAAGGAC	EF577880	EF577881	EF577882	EF577883	EF577884	EF577885	EF577886	EU503378	EU503379	EU503380
8_02	AGGACCCGTCGCTCACCTTC	CGCGAGCGGCCCGTGAAGAT	EF578024	EF578025	EF578026	---	EF578027	---	EF578028	---	---	---
8_03	CCAGCTTCAGCGAGATCGTG	CCCTTCAGATTCAAGCAACC	EF578360	EF578361	EF578362	EF578363	EF578364	EF578365	EF578366	---	---	---
8_04	GTGGATTCTGGAGGGAACGC	GCATTGTGGGCAGCCATATT	EF577683	EF577684	EF577685	EF577686	EF577687	EF577688	EF577689	---	---	---
8_05	TCCATTGCCCAGAAGTATGA	TCTCCATTGCGATGAGTCCT	EF577708	EF577709	EF577710	EF577711	EF577712	EF577713	EF577714	---	---	---
8_06	TGGGCTGAGGCTGTAGGGAT	GTCTTCGGCTCGCCATCGTG	EF578372	EF578373	EF578374	---	---	EF578375	---	---	---	---
8_07	CTGATGTTGATTGGAGTTATGG	AGGATGTCGTGGCTTGAGTGTA	EF577539	EF577540	EF577541	EF577542	EF577543	EF577544	---	---	---	---
8_08	AGAGGCAGCAGATAAGAACCAG	CCATAGGACGCATTTGTGAGAA	EF577587	EF577588	EF577589	EF577590	EF577591	EF577592	EF577593	---	---	---
8_09	CGATGAAGGGCGACAACCAG	CTCCTTCCGGTGATCTCGTG	EF578088	EF578089	EF578090	---	EF578091	EF578092	EF578093	---	---	---
8_10	CTCCTGATGATGATTGTGAG	GAAGGTGACCACGGGCAAGT	EF578048	EF578049	EF578050	EF578051	EF578052	EF578053	EF578054	---	---	---
8_11	GGACGAGGTGTGGCGGGACT	TTCAGCTTCCAACTCCATTG	EF578055	EF578056	EF578057	EF578058	EF578059	EF578060	EF578061	---	---	---

8_12	GCGCTTAAAGATAGGCTGAT	GGGCTATTGAGATTGATGAG	EF577860	EF577861	EF577862	EF577863	EF577864	EF577865	EF577866	EU503381	EU503382	EU503383
9_01	GAGCTTAATCTCCCAAATTC	ATGATGCCCGATCTCGAAAG	EF577846	EF577847	EF577848	EF577849	EF577850	EF577851	EF577852	EU503384	EU503385	EU503386
9_02	GGAATGCGACAAGAATTTGC	TCGGCTGGACAATGGCAAAG	EF577887	EF577888	EF577889	EF577890	EF577891	EF577892	EF577893	EU503387	EU503388	EU503389
9_03	CCATCAAGGATACGCAAGAG	CCCGTCATATCCATAACCTT	EF577874	EF577875	EF577876	---	EF577877	EF577878	EF577879	EU503390	EU503391	EU503392
9_04	CGGCTATCCAATCCTTGAAG	AGTTCTGCCTCTAGTTCGTC	EF577933	EF577934	EF577935	EF577936	EF577937	EF577938	EF577939	EU503393	EU503394	EU503395
9_05	ACTTTCTATTGCGAGCTATG	ACCCAATCTTGTCGGCTTTC	EF578413	EF578414	EF578415	EF578416	EF578417	EF578418	EF578419	---	---	---
9_06	GCGCAGGCAAATAAACCGAC	ACCGGTAGCTGTCACCATTC	EF577953	EF577954	EF577955	EF577956	---	EF577957	EF577958	EU503396	EU503397	EU503398
9_07	CCCGTCAATACTGTACTTGC	AGGCCCGAGTAAGGTACTGC	EF578390	EF578391	EF578392	EF578393	EF578394	EF578395	EF578396	---	---	---
9_08	GTCATCATGGCATCCGACAC	AGTATGGCGGGTAGATGGTC	EF577940	EF577941	EF577942	EF577943	EF577944	EF577945	EF577946	EU503399	EU503400	EU503401
9_09	GATAGCTGGAGCTGTTACTT	CTCAGGCCTTGTTACAT	EF577697	EF577698	EF577699	EF577700	EF577701	EF577702	EF577703	---	---	---
9_10	CACGCACAACAACCCGAATG	TGGATGGTTAATGCCGGTCT	EF578376	EF578377	EF578378	EF578379	EF578380	EF578381	EF578382	---	---	---
10_01	CTTCTGGAAGGTCCTTCAAG	TGGATCCACTGGCTGGTAAC	EF578104	EF578105	EF578106	EF578107	EF578108	EF578109	EF578110	---	---	---
10_02	TGAAAGTGTTCCAGCAGGAG	ATCTGCCGTTTTTATTTCAG	EF578122	EF578123	EF578124	EF578125	---	---	---	---	---	---
10_03	AGAATCCAAATGCCGCTCTG	AATTCCTTGTACGCCTGCTG	EF578118	EF578119	EF578120	EF578121	---	---	---	---	---	---
10_04	TTCTGTATGCTGCCAAAGAG	TAATTGCATGGGCTCCAACT	EF577947	EF577948	EF577949	EF577950	---	EF577951	EF577952	EU503402	EU503403	EU503404
10_05	GGCCCGCAGCAACCGTTATC	TCGGCGTGAACTCGGAGAAG	EF578133	EF578134	EF578135	EF578136	EF578137	EF578138	---	---	---	---
10_06	TGGACTGCAGGTTGGATCTG	TAAATCAGCCCAGGGAAGAG	EF577894	EF577895	EF577896	EF577897	EF577898	EF577899	EF577900	EU503405	EU503406	EU503407
10_07	TCTTCTGGGCCCTAATTTATC	TGGCAGATTCGTATGGAGTTG	EF577906	EF577907	EF577908	EF577909	EF577910	EF577911	EF577912	EU503408	EU503409	EU503410
10_08	TGCATTTTGGAGAGAGCATC	ATGTGACCACCACGCGATCT	EF578153	EF578154	EF578155	EF578156	EF578157	EF578158	EF578159	---	---	---
10_09	TCTGGCATCGTTTGTGTTCT	ATTTTCCAGGCTGCTGAATC	EF577901	EF577902	EF577903	EF577904	---	---	EF577905	EU503411	EU503412	EU503413
10_10	GAGCGTCTAATGCGATTTGC	AGGCAATGATCTTATCGTTC	EF577704	EF577705	EF577706	EF577707	---	---	---	---	---	---
10_11	AGACATGGAACAGAAGCTCC	CATCAGAACAACATTATGGC	EF577615	EF577616	EF577617	EF577618	EF577619	EF577620	EF577621	---	---	---
11_01	ACGGAAGTTCTGCTTGTTGT	AAGTCTTTTCTCGGCTTCTC	EF578029	EF578030	EF578031	EF578032	---	EF578033	EF578034	EU503426	EU503427	EU503428
11_02	AGTTGAGCTGCCATTGACAG	CCCGTAAGCCTAACCTTGAC	EF578035	EF578036	EF578037	EF578038	EF578039	EF578040	EF578041	EU503429	EU503430	EU503431
11_03	CACACCGACGTCTACTTCTG	TCAGCAAGTACCTAAATTATC	AF148569	AF148577	AF148579	AF148589	AF148598	AF148597	AF148600	---	---	---
11_04	CACACCGACGTCTACTTCTG	CCACCGTTGGTCATCTCAAT	AF148603	AF148611	AF148613	AF148623	AF148632	AF148631	AY792581	---	---	---

11_05	ACTTTGGGTGGCCGCTCTTC	TCCGCCTTATCCTTGTCAAC	EF578068	EF578069	EF578070	EF578071	EF578072	EF578073	EF578074	---	---	---
11_06	GCGCTTTGATTGCATCGTTG	GAAGCGTTTTCCTCATTTAC	EF578094	EF578095	EF578096	EF578097	EF578098	EF578099	---	---	---	---
11_07	CGCGTGAACTTACTGCTGTG	TGCTGGCCCTGCTGATGAAC	EF578139	EF578140	EF578141	EF578142	EF578143	EF578144	EF578145	---	---	---
11_08	CATGCAGGCTGATGAGG	CTTCTTCTGGGTAATGCTCT	EF577785	EF577786	EF577787	EF577788	EF577789	EF577790	EF577791	---	---	---
11_09	GTCATTGAGGCTGGATTACC	ATCCTCTTCATCGCCTTCAG	EF578100	EF578101	EF578102	EF578103	---	---	---	---	---	---
11_10	TTTCCAGCCAACCATTAGAG	GCAGACGCAGCTAGTTGTTC	EF577690	EF577691	EF577692	EF577693	EF577694	EF577695	EF577696	---	---	---
11_11	CAAGGGTTTTGTTGCACAAG	TGACAGAATGCTGATACGCT	EF577719	EF577720	EF577721	EF577722	EF577723	EF577724	EF577725	---	---	---
11_12	CGCTTCAGTTAAGGCAATAG	TTGAAGTATGGCAAGACAAG	EF578126	EF578127	EF578128	EF578129	EF578130	EF578131	EF578132	---	---	---
11_13	AGTGACCAAGTCCCGAGAAC	TCACAAATTGACAGGTTCCC	EF578383	EF578384	EF578385	EF578386	EF578387	EF578388	EF578389	---	---	---
12_01	AGGCAATCCGGTTACACCTG	AGCAGGGCAAAGACTTGATG	EF578111	EF578112	EF578113	EF578114	EF578115	EF578116	EF578117	---	---	---
12_02	CATTTTACATAGCTGGGGAGTC	ACCATACAAGGAACTCCACTG	EF577552	EF577553	EF577554	EF577555	EF577556	EF577557	EF577558	---	---	---
12_03	CTACATCGTGTTGGACGCAC	CTGCAACAATGCGACTTTTC	EF578062	EF578063	EF578064	---	EF578065	EF578066	EF578067	---	---	---
12_04	GTGCCAGACACACTCTACCT	CTGTGCGGCTCTAAGGAATC	EF577913	EF577914	EF577915	EF577916	EF577917	EF577918	EF577919	EU503414	EU503415	EU503416
12_05	GAAAAGCAGGTGCCAATCAT	TCCCAGCCAATGGTGAAGTG	EF577920	EF577921	EF577922	EF577923	EF577924	EF577925	EF577926	EU503417	EU503418	EU503419
12_06	GAAGGGGGAGAAGTTCTACC	AATGATGGAACCGCTTGCAC	EF578420	EF578421	EF578422	EF578423	EF578424	EF578425	EF578426	---	---	---
12_07	TGAAGTTGCCGCCCAGACAG	TCGGTCTCGGAATTGATCTC	EF577927	EF577928	EF577929	EF577930	---	EF577931	EF577932	EU503420	EU503421	EU503422
12_08	CCTTTGCTGAACTGGAAGAG	GCTTGACAAATCCCCGTATG	EF577959	EF577960	EF577961	EF577962	EF577963	EF577964	EF577965	EU503423	EU503424	EU503425
12_09	GCTGGAGATAGCTGACAAGT	GGTCGAAGGTTAGGGTTC	EF577726	EF577727	EF577728	EF577729	---	---	---	---	---	---

* corresponding to gene's ID defined by authors in Additional data files 1 and 2.

† not available
